# Integrating preexposure prophylaxis delivery in routine family planning clinics: A feasibility programmatic evaluation in Kenya

**DOI:** 10.1371/journal.pmed.1002885

**Published:** 2019-09-03

**Authors:** Kenneth K. Mugwanya, Jillian Pintye, John Kinuthia, Felix Abuna, Harrison Lagat, Emily R. Begnel, Julia C. Dettinger, Grace John-Stewart, Jared M. Baeten, for the PrEP Implementation for Young Women and Adolescents (PrIYA) Program

**Affiliations:** 1 Department of Global Health, University of Washington, Seattle, Washington, United States of America; 2 Department of Obstetrics/Gynecology, Kenyatta National Hospital, Nairobi, Kenya; 3 University of Washington-Kenya, Nairobi, Kenya; 4 Department of Epidemiology, University of Washington, Seattle, Washington, United States of America; 5 Department of Medicine, University of Washington, Seattle, Washington, United States of America; 6 Department of Pediatrics, University of Washington, Seattle, Washington, United States of America; University of California, San Francisco, UNITED STATES

## Abstract

**Background:**

Young women account for a disproportionate fraction of new HIV infections in Africa and are a priority population for HIV prevention, including implementation of preexposure prophylaxis (PrEP). The overarching goal of this project was to demonstrate the feasibility of integrating PrEP delivery within routine family planning (FP) clinics to serve as a platform to efficiently reach at-risk adolescent girls and young women (AGYW) for PrEP in HIV high-burden settings.

**Methods and findings:**

The PrEP Implementation in Young Women and Adolescents (PrIYA) program is a real-world implementation program to demonstrate integration of PrEP delivery for at-risk AGYW in FP clinics in Kisumu, Kenya. Between November 2017 and June 2018, women aged 15 to 45 from the general population seeking FP services at 8 public health clinics were universally screened for HIV behavioral risk factors and offered PrEP following national PrEP guidelines. We evaluated PrEP uptake and continuation, and robust Poisson regression methods were used to identify correlates of uptake and early continuation of PrEP, with age included as a one-knot linear spline. Overall, 1,271 HIV-uninfected women accessing routine FP clinics were screened for PrEP; the median age was 25 years (interquartile range [IQR]: 22–29), 627 (49%) were <24 years old, 1,026 (82%) were married, more than one-third (34%) had partners of unknown HIV status, and the vast majority (*n* = 1,200 [94%]) reported recent condom-less sex. Of 1,271 women screened, 278 (22%) initiated PrEP, and 114 (41%) returned for at least one refill visit after initiation. PrEP uptake was independently associated with reported male-partner HIV status (HIV-positive 94%, unknown 35%, HIV-negative 8%; *p* < 0.001) and marital status (28% unmarried versus married 21%; *p* = 0.04), and a higher proportion of women ≥24 years (26%; 191/740) initiated PrEP compared to 16% (87/531) of young women <24 years (*p* < 0.001). There was a moderate and statistically non-significant unadjusted increase in PrEP uptake among women using oral contraception pills (OCPs) compared to women using injectable or long-acting reversible contraception methods (OCP 28% versus injectable/implants/intrauterine devices [IUDs] 18%; *p* = 0.06). Among women with at least one post-PrEP initiation follow-up visit (*n* = 278), no HIV infection was documented during the project period. Overall, continuation of PrEP use at 1, 3, and 6 months post initiation was 41%, 24%, and 15%, respectively. The likelihood for early continuation of PrEP use (i.e., return for at least one PrEP refill within 45 days post initiation) was strongly associated with reported male-partner HIV status (HIV-positive 67%, -negative 39%, unknown 31%; overall effect *p* = 0.001), and a higher proportion of women ≥24 years old continued PrEP at 1 month compared with young women <24 years old (47% versus 29%; *p* = 0.002). For women ≥24 years old, the likelihood to continue PrEP use at 1 month post initiation increased by 3% for each additional year of a woman’s age (adjusted prevalence ratio [PR]: 1.03; 95% confidence interval [CI]: 1.01–1.05; *p* = 0.01). In contrast, for women <24 years old, the likelihood of continuing PrEP for each additional year of a woman’s age was high in magnitude (approximately 6%) but statistically non-significant (adjusted PR: 1.06; 95% CI: 0.97–1.16; *p* = 0.18). Frequently reported reasons for discontinuing PrEP were low perceived risk of HIV (25%), knowledge that partner was HIV negative (24%), experiencing side effects (20%), and pill burden (17%). Study limitations include lack of qualitative work to provide insights into women’s decision-making on PrEP uptake and continuation, the small number of measured covariates imposed by the program data, and a nonrandomized design limiting definitive ascertainment of the robustness of a PrEP-dedicated nurse-led implementation strategy.

**Conclusions:**

In this real-world PrEP implementation program in Kenya, integration of universal screening and counseling for PrEP in FP clinics was feasible, making this platform a potential “one-stop” location for FP and PrEP. There was a high drop-off in PrEP continuation, but a subset of women continued PrEP use at least through 1 month, possibly indicating further reflection or decision-making on PrEP use. Greater efforts to support PrEP normalization and persistence for African women are needed to help women navigate their decisions about HIV prevention preferences as their reproductive goals and HIV vulnerability evolve.

## Abstract 2

Kenneth Mugwanya and colleagues report on implementation of a pre-exposure prophylaxis programme for young women and adolescent girls at risk of HIV infection, in Kenya.

## Author summary

**Why was this study done?:**

- Adolescent girls and young women (AGYW) in Africa are disproportionately affected by HIV infections because of cultural, structural, biological, and behavior factors.
- Preexposure prophylaxis (PrEP), as a highly potent and recommended discreet user-controlled HIV prevention strategy, has the potential to substantially reduce new HIV infections in African women if delivered with high coverage and if used with sufficient adherence.
- However, data are limited on “real-world” implementation approaches to efficiently reach at-risk women who may benefit from PrEP.
- Family planning (FP) clinics provide care to young women at risk for acquiring HIV and have in-built staffing, supply chain, and HIV testing access, which could contribute to more efficient PrEP implementation with less cost, but no study has evaluated this model in “real-world” settings.

**What did the researchers do and find?:**

- We conducted a pilot open-label, “real-world” implementation program to evaluate the feasibility of integrating PrEP delivery into routine FP clinics to reach HIV at-risk young women.
- General-population women accessing FP services were universally screened for HIV behavior risk factors and were counseled for PrEP by program-dedicated nurses embedded in 8 public health FP clinics in a high–HIV-prevalence region in Kenya.
- We found that FP clinics can be an effective platform to efficiently reach HIV at-risk women who may benefit from PrEP.
- PrEP screening was feasible, and 22% of the general population of women took PrEP home. There was a high drop-off in early PrEP continuation (41% PrEP continuation at 1 month) particularly among AGYW <24 years old, but a subset of women persisted on PrEP (25% at month 3 and 15% at month 6), with higher continuation among women with HIV-positive partners, possibly indicating further reflection or decision-making on PrEP use.
- Women’s perceived risk for HIV, including having an HIV-positive partner, was an important driver of initiation and continuation on PrEP.

**What do these findings mean?:**

- To our knowledge, this project provides the first demonstration of “real-world” delivery of PrEP for at-risk AGYW integrated into FP clinics in Africa, a priority population for HIV prevention.
- These findings demonstrate that it may be feasible to integrate PrEP delivery in public health FP clinics, making this a platform potential “one-stop” location for FP and PrEP. With expanding PrEP awareness, uptake and continuation are likely to increase among women at risk.
- Evidence from this work will inform next steps for wider delivery of PrEP and next-generation PrEP formulations (e.g., multipurpose HIV prevention and contraception technologies) in FP clinics, not only in Kenya but in other resource-limited settings globally.

## Introduction

Young women in HIV high-burden settings are a priority population for HIV prevention because they account for a disproportionate fraction of new HIV infections [1]. Preexposure prophylaxis (PrEP) is a safe and highly potent intervention when taken daily and has the potential to substantially reduce new infections if delivered with sufficient coverage to populations with greatest HIV prevention needs [2–5]. As PrEP implementation gradually comes to scale in many HIV high-burden regions, care settings routinely accessed by young women could be leveraged as a platform for reaching this important at-risk group. Family planning (FP) clinics are a particularly attractive platform for integrating PrEP delivery because FP providers are uniquely positioned to counsel on PrEP as they already counsel women on sexual health services. Women also are routinely screened for sexual behavior and HIV risk factors in FP clinics, and PrEP screening could be integrated efficiently within this context. Importantly, PrEP can safely be used with commonly used hormonal contraceptives with no bilateral drug-drug interactions [6,7].

In September 2015, the World Health Organization (WHO) recommended PrEP as a prevention option for persons at high risk of HIV acquisition [8]. Subsequently, in July 2016, the Kenya Ministry of Health (MOH) released guidelines that recommended PrEP for all HIV-uninfected persons with substantial ongoing risk of HIV infection, including adolescent girls and young women (AGYW) as priority persons [9]. In Kenya and many other African settings, FP clinics already incorporate HIV prevention services such as HIV counseling and testing. However, limited data are available on implementation approaches on how to efficiently reach and counsel women for PrEP in these settings, particularly young women. Here, we report on uptake and early continuation of PrEP in a real-world implementation program integrated in routine FP clinic settings in Kenya.

## Methods

### Program design and context

The PrEP Implementation in Young Women and Adolescents (PrIYA) program was an implementation program to deliver PrEP to young women at substantial risk of HIV in Kisumu Kenya. The protocol (S1 File) and conduct of this project were fully compliant with the relevant Kenya MOH regulations and were approved by the Human Subjects Division of the University of Washington, the Kenyatta National Hospital Ethical Review Committee, and the Kisumu County administration and facility managers. Women provided verbal informed consent as is routinely done for standard of care services. This project is reported as per the Strengthening of the Reporting of Observational Studies in Epidemiology (STROBE) guideline (S1 Checklist).

The overall goal of the project was to demonstrate the feasibility of integrating PrEP delivery in public health maternal and child health (MCH) and FP clinics. PrIYA is part of the larger Determined, Resilient, Empowered, AIDS-free, Mentored, and Safe women (DREAMS) Innovation Challenge funded by the President's Emergency Plan For AIDS Relief (PEPFAR) managed by JSI Research & Training Institute. Following the release of the Kenyan national guidelines that recommended PrEP as part of standard of care HIV prevention [9], the Kenya MOH developed a national PrEP implementation framework and service provider toolkit in 2017 [10]. Kenya officially launched PrEP rollout nationally in May 2017. A preparatory phase for the PrIYA program commenced in July 2017, with full-scale implementation starting in November 2017. Between November 2017 and June 2018, in collaboration with the Kisumu County Government and the Kenya National AIDS and Sexually Transmitted Infection (STI) Control Programme (NASCOP), PrIYA operationalized PrEP counseling and delivery in 16 facilities (including 8 facilities with FP clinics as a delivery point for PrEP) in Kisumu County, Kenya. This region has an HIV prevalence of up to 28% among young women [11–13].

### Population and settings

The current report details the operationalization of PrEP implementation integrated in routine FP clinics. The program targeted all HIV-uninfected women of reproductive age, 15 to 45 years old, seeking routine FP services in 8 high-volume public health FP clinics in Kisumu County, Kenya. Implementation clinics were selected based on high volume and geography in consultation with the Kisumu County health authorities: at pre-implementation assessment, the monthly volumes of women newly accessing FP services at these clinics were approximately <50, 50–100, and >100 at 1, 5, and 2 of the clinics, respectively. One clinic was classified as rural, 4 as semi-urban, and 2 as urban.

### Implementation strategies and program activities

The primary implementation strategy was a PrEP-dedicated nurse-led delivery of counseling about HIV risk and provision of PrEP. Newly hired nurses were trained on HIV risk assessment, counseling, and PrEP provision using a 2-day case-based interactive Kenya MOH PrEP curriculum, and knowledge gain was assessed by pre- and post-test. Nurses only performed HIV risk counseling and provision of PrEP but did not participate in delivery of FP services. At nearly all of the 8 clinics, women first completed other services, including HIV testing, and were then referred to a PrEP-dedicated nurse. Specifically, women of reproductive age accessing FP services were universally counseled by a PrEP-program–dedicated nurse for HIV behavioral risk factors and willingness to consider PrEP for HIV prevention. Screening was conducted according to the Kenya PrEP national guidelines [9], guided by a Kenya MOH risk assessment screening tool (RAST) modified to include women’s self-assessed reasons for choosing or declining PrEP (S2 File); the tool was used only as a guide but not as a scoring tool for ruling in or out potential users. Behavioral factors defined by the Kenya PrEP guidelines to indicate a substantial ongoing risk of acquiring HIV include the following: (a) inconsistent or no condom use in the last 6 months; (b) having a high-risk sex partner(s) of unknown HIV status; (c) engaging in transactional sex; (d) history of ongoing intimate partner violence (IPV) and gender-based violence (GBV); (e) recent bacterial STIs, self-reported or etiologically diagnosed; (f) recurrent use of postexposure prophylaxis; (g) recurrent sex under the influence of alcohol and/or recreational drugs; (h) injection drug use with shared needles and/or syringes; and (i) having an HIV-positive partner [9]. Interested and medically eligible women were provided same-day PrEP initiation by the nurse.

### Follow-up and PrEP medication

Consistent with the programmatic nature of this work, visit schedules reflected approaches used in FP clinics to permit seamless integration in routine services in Kenya. Women initiated on PrEP were followed as per the Kenya national guidelines for PrEP, which include initiation, month 1, and then 3 monthly visits for clinical review. However, in order to cautiously manage PrEP commodities, most participants are dispensed monthly PrEP refills. PrEP commodities were supplied from the Kenya Medical Supply Authority.

### Data collection

For this implementation program, patient medical records were captured on standardized data collection tools including the MOH clinical encounter form for the clinical provision of PrEP for all populations in Kenya. Program data including women’s demographics, behavioral-risk characteristics, reported partner HIV status, PrEP uptake, self-reported adherence to PrEP, and adverse events were abstracted daily by program nurses. Continuation and adherence on PrEP was assessed by self-report and PrEP refill records at the clinic as well as through follow-up phone calls to ascertain PrEP continuation status and reasons for discontinuing PrEP. All data were entered into passward-protected tablets daily and uploaded to web-based encrytped Research Electronic Data Capture (REDCap) servers [14]. Internal quality control reports were run weekly to monitor program progress and discussed with clinics throughout program implementation. All relevant data underlying this manuscript are included in S1 Data.

### Key program outcomes

The co-primary program implementation outcomes were the number of women screened for HIV behavioral risk factors (reach) and the number of women who initiated PrEP (uptake or adoption). Additional key outcomes were continuation on PrEP, behavioral risk profile of women, male-partner HIV status, reasons for declining PrEP, self-assessed adherence to PrEP, contraception method use, and correlates of PrEP initiation and early continuation. Early PrEP continuation was defined as return to clinic and PrEP refill within 45 days post initiation. We also summarized PrEP continuation rates at 3 and 6 months post initiation using available data from regularly scheduled follow-up visits.

### Analysis

Categorical variables were summarized as frequencies, and continuous measures were summarized as medians and ranges, as appropriate. Baseline demographic and behavioral factors characterized the HIV risk profiles of women screened for PrEP. Separately, we evaluated for the correlates of PrEP uptake and early PrEP continuation using Poisson regression methods with robust standard errors to generate prevalence ratios (PRs) and 95% confidence intervals (CIs) accounting for clinic clustering, an approach used when the occurrence of the outcome is high (>10%) [12,15]. For the correlates of PrEP uptake, age, marital status, reported male-partner HIV status, and contraception method were considered a priori to have substantial influence on uptake and were subsequently included in the multivariate analysis. However, because of sparse data involving the contraception methods variable (≤10% frequency of use for some contraception methods), contraception use was only evaluated at the unadjusted level. Age was fit as linear spline with a single knot at age 24, reflecting rate of change in outcome for each additional year of a woman’s age within each age group. To aid meaningful interpretation, we also present key outcomes stratified by age groups (i.e., ≥24 versus <24 years). A similar approach was used for the analysis of early PrEP continuation. Baseline HIV risk behavior covariates were evaluated for their independent effects on PrEP continuation if they had a *p* ≤ 0.2 in the unadjusted analysis. Self-reported adherence and reasons for discontinuing PrEP were presented overall and stratified by male-partner HIV status. Statistical analyses were conducted in Stata version 15 (Stata Corporation, College Station, TX).

## Results

### General characteristics

Overall, we screened 1,271 HIV-uninfected women for behavioral risk factors and willingness to initiate PrEP among women accessing FP services in 8 clinics in Kisumu County, Kenya. The median age of women screened was 25 years (interquartile range [IQR]: 22–29), 8% (105/1,271) were <20 years old, and 49% (627/1,271) were <24 years old; 82% (1,026/1,271) were married, and more than a third (427/1,271) of the women did not know their male partners’ HIV status (Table 1). The vast majority of women (*n =* 1,200 [94%]) reported recent condom-less sex. Most women (1,121 [92%]) reported using some form of contraception at baseline; the most frequently used FP methods were injectable (56%), implants (31%), and oral contraception pill (OCP) (5%), with 3% using intrauterine devices (IUDs) and 2% condoms alone. Notably, 75% (45/60) of those who used OCPs were women ≥24 years old.

**Table 1:** Baseline characteristics of women screened for PrEP, by male-partner HIV status (*N =* 1,271) (*N* [%] or median [IQR]).

	Reported male-partner HIV status			
Characteristics	Overall (*N =* 1,271)	Negative (*n =* 772; 61%)	Unknown (*n =* 427; 34%)	Positive (*n =* 65; 5%)
***Age***	25 (22–29)	24.0 (22.0–29.0)	24.0 (21.0–29.0)	30.0 (25.0–35.0)
***Age category (years****)*				
<20	105 (8.3)	62 (8.0%)	36 (8.4%)	4 (6.2%)
20–24	522 (41.1)	333 (43.1%)	178 (41.7%)	10 (15.4%)
25–29	356 (28.0)	221 (28.6%)	119 (27.9%)	13 (20.0%)
30–34	172 (13.5)	103 (13.3%)	50 (11.7%)	19 (29.2%)
≥35	116 (9.1)	53 (6.9%)	44 (10.3%)	19 (29.2%)
***Marital status***				
Married/cohabiting	1,026 (81.8)	648 (84.9)	318 (75.5)	60 (93.8)
Not married/cohabiting	229 (18.2)	115 (15.1)	103 (24.5)	4 (6.2)
Missing	16 (1.3%)	9 (1.2%)	6 (1.4%)	1 (1.5%)
***Marriage type (n = 1*,*011)***				
Polygamous	956 (94.6)	620 (97.0)	287 (91.7)	49 (83.1)
Monogamous	55 (5.4)	19 (3.0)	26 (8.3)	10 (16.9)
**Clinical characteristics**				
***Any FP method***	1,121 (91.8)	706 (93.6)	375 (91.9)	36 (69.2)
***Contraceptive type***^***1***^ ***(n = 1*,*117)***				
Injectable	627 (55.9)	391 (55.4)	216 (57.6)	19 (52.8)
Implant	345 (30.8)	224 (31.7)	104 (27.7)	14 (38.9)
OCP	60 (5.4)	34 (4.8)	26 (6.9)	0 (0.0)
IUD	37 (3.3)	26 (3.7)	11 (2.9)	0 (0.0)
Condoms only	27 (2.4)	16 (2.3)	9 (2.4)	2 (5.6)
Other method^2^	18 (1.6)	11 (1.6)	6 (1.6)	1 (2.8)
Missing	7 (0.6)	4 (0.6)	3 (0.8)	0 (0.0)
**Behavioral risk factors in last 6 month**				
Had sex without a condom	1,200 (94.4)	736 (95.3)	401 (93.9)	57 (87.7)
Engaged in sex in exchange for money/favors	13 (1.0)	4 (0.5)	5 (1.2)	4 (6.2)
Diagnosed with or treated for an STI	14 (1.1)	5 (0.6)	8 (1.9)	1 (1.5)
Forced to have sex	21 (1.7)	4 (0.5)	11 (2.6)	6 (9.2)
Experienced IPV	46 (3.6)	11 (1.4)	29 (6.8)	6 (9.2)
Shared needles while engaging in IVD	0 (0.0)	0 (0.0)	0 (0.0)	0 (0.0)
Recurrent PEP use	2 (0.2)	1 (0.1)	1 (0.2)	0 (0.0)

^1^Among women who reported using at least one method of FP (*n =* 1,117).

^2^Other methods specified included locational amenorrhea, withdrawal, natural methods, and tubal ligation.

**Abbreviations:** FP, family planning; IPV, intimate partner violence; IQR, interquartile range; IUD, intrauterine device; IVD, intravenous drug use; OCP, oral contraception pill; PEP, postexposure prophylaxis; PrEP, preexposure prophylaxis; STI, sexually transmitted infection

### PrEP uptake and correlates of PrEP uptake

**Fig 1:**
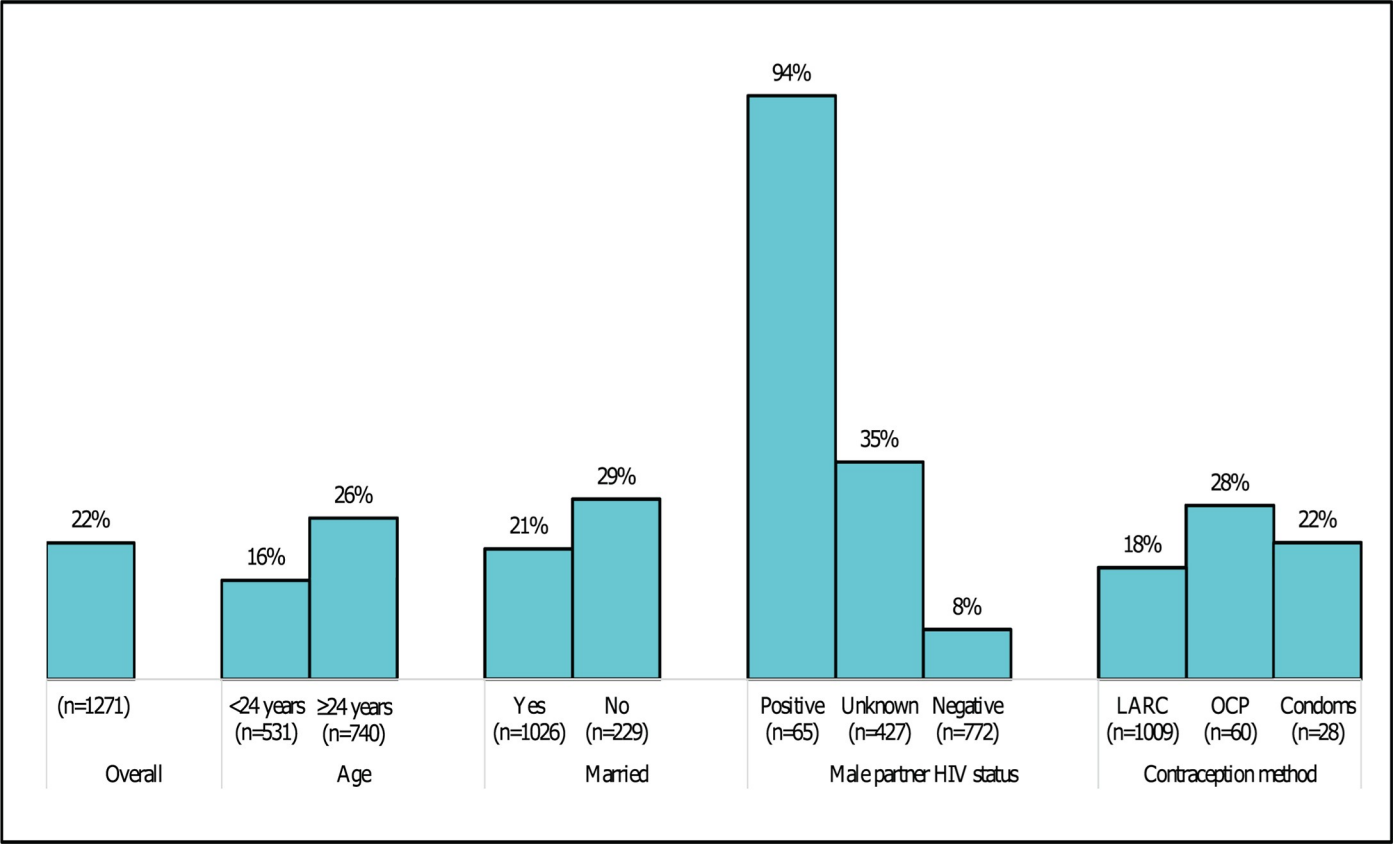
PrEP uptake stratified by key baseline covariates. LARC, Long-acting reversible contraception; OCP, oral contraception pill; PrEP, preexposure prophylaxis.

Of 1,271 HIV-uninfected women universally screened and counseled for PrEP, 278 (22%) initiated PrEP overall (Fig 1)—87 out of 531 (16%) among women <24 years old versus 191 out of 740 (26%) for women ≥24 years old (*p* < 0.001). Women who initiated PrEP had behavioral risk factors for HIV as defined by the Kenya national PrEP program guidelines; the most frequent HIV behavioral risk factors were recent condom-less sex (264/278; 95%), having a male partner of unknown HIV status (151/278; 54%), and having an HIV-positive male partner (61/278; 22%). History of IPV or being forced to have sex in the prior 6 months was reported by <10% of women, respectively.

Overall, age, reported male-partner HIV status, and marital status were baseline factors independently associated with PrEP uptake (Table 2). All but 4 of 65 (94%) women with HIV-positive partners initiated PrEP compared to 35% (151/427) of women with partners of unknown HIV status and 8% (65/722) for women with negative partners (*p* < 0.001). Notably, women with a positive male partner tended to be older compared to women with partners either negative or of unknown status (median age: 30 versus 24 years; *p* < 0.001). Similarly, a higher proportion of women ≥24 years old (26%; 191/740) initiated PrEP compared to 16% (87/531) among women <24 years old (*p* < 0.001). For women ≥24 years, the likelihood of initiating PrEP increased by about 3% for each additional year of a woman’s age (adjusted PR: 1.03; 95% CI: 1.0–1.05; *p* < 0.001). In contrast, for women in the group <24 years old, the likelihood of initiating PrEP for each additional year of age was approximately 6% but statistically non-significant (adjusted PR: 1.05; 95% CI: 0.99–1.12; *p =* 0.12). PrEP initiation was also independently higher among unmarried women compared to married women (28% versus 21% for married; *p =* 0.03). There was a moderate and statistically non-significant higher likelihood for PrEP uptake among women using OCP compared to women using injectable or long-acting reversible hormonal contraceptive (i.e., implants and IUD) methods (28% versus 18%; *p =* 0.06). Among women who declined PrEP but had at least one behavioral HIV risk factor as defined by national PrEP guidelines (*n =* 954), the most frequently reported reasons for not initiating PrEP were low perceived risk of acquiring HIV (43%), knowledge that their male partner was HIV negative (47%, among HIV-uninfected women), needing to consult male partner (21%), and pill burden (13%). Fear of IPV or side effects were reported by less than 5% of women who did not initiate PrEP. Women with partners of unknown HIV status who declined PrEP (*n =* 276) frequently reported needing to consult their partners (41%), low perceived risk of HIV (24%), and pill burden (21%) as reasons for declining PrEP. Of the 4 women with HIV-positive partners who chose not initiate PrEP, one had a partner who was virally suppressed, one needed to consult her partner, one had concerns about pill burden, and no reason was recorded for the fourth woman.

**Table 2:** Correlates of PrEP uptake (*N* = 1,271).

Demographic characteristics	Accepted PrEP*		Unadjusted		Multivariable	
	No (*n =* 993)	Yes (*n =* 278)	Crude PR (95% CI)	*p*-Value	Adjusted PR (95% CI)	*p*-Value†
Age (years)**						
<24	444 (83.6)	87 (16.4)	1.05 (0.98–1.12)	0.11	1.05 (0.99–1.12)	0.12
≥24	549 (74.2)	191 (25.8)	1.03 (1.02–1.05)	**<0**.**001**	1.03 (1.02–1.05)	**<0**.**001**
**Marital status**						
Not married/cohabiting	164 (71.6)	65 (28.4)	1.39 (1.00–1.93)	**0**.**05**	1.62 (1.05–2.49)	**0**.**03**
Married/cohabiting	816 (79.5)	210 (20.5)	Ref		Ref	
**Marriage type (*N =* 1,011)**						
Polygamous	18 (32.7)	37 (67.3)	3.85 (2.77–5.35)	**<0**.**001**		
Monogamous	789 (82.5)	167 (17.5)	Ref			
**Partner HIV status**						
Positive	4 (6.2)	61 (93.8)	11.15 (7.86–15.81)	**<0**.**001**	9.67 (7.00–13.35)	**<0**.**001**
Unknown	276 (64.6)	151 (35.4)	4.20 (3.00–5.88)	**<0**.**001**	3.90 (2.74–5.56)	
Negative	707 (91.6)	65 (8.4)	Ref		Ref	
**Contraceptive type (*N =* 1,096)**						
OCP	43 (71.7)	17 (28.3)	1.55 (0.99–2.44)	0.06		
Condoms only	21 (77.8)	6 (22.2)	1.22 (0.78–1.92)	0.39		
Injectable/implants/IUD	825 (81.8)	184 (18.2)	Ref			

Bolding denotes statistical significance.

*Row percent.

**†**Adjusted *p*-value for overall effects; for age, this *p*-value is for slope within the respective age group.

**Linear spline with one knot at 24 years.

**Abbreviations:** IUD, intrauterine device; OCP, oral contraception pill; PR, prevalence ratio; PrEP, preexposure prophylaxis.

### PrEP continuation

**Fig 2:**
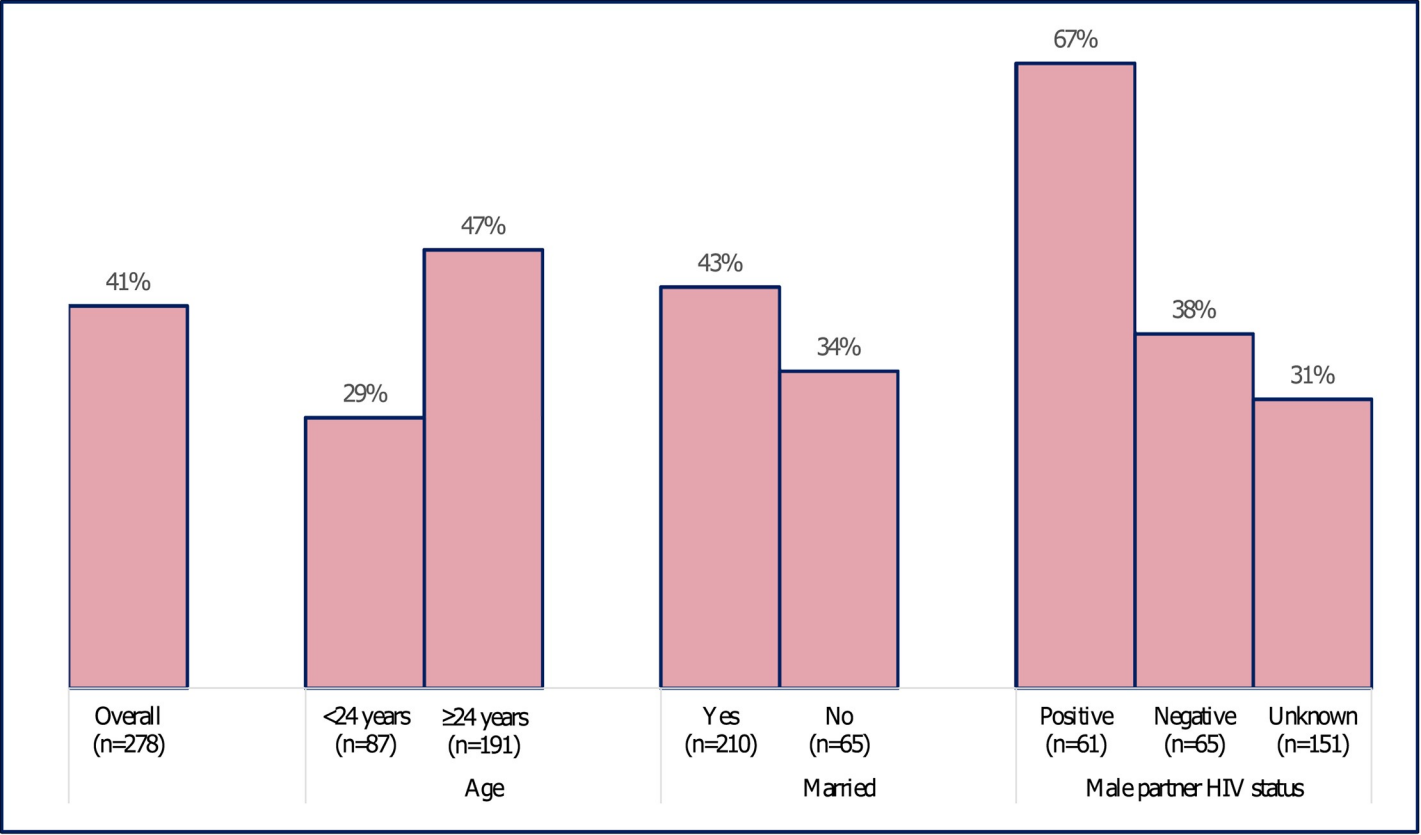
PrEP continuation at month 1 stratified by key baseline covariates. PrEP, preexposure prophylaxis.

Overall, among all women who initiated PrEP (Table 3), 114 out of 278 (41%) returned to collect at least one PrEP refill within 45 days post initiation (Fig 2; continuation for key subgroups), 28 out of 278 (10%) returned to report discontinuing PrEP, and 136 out of 278 (49%) did not return for a PrEP refill.

**Table 3:** Correlates of 1-month PrEP continuation (*N* = 278).

	PrEP continuation at month 1*		Unadjusted		Multivariable	
Demographic characteristics	No (*n* = 164)	Yes (*n* = 114)	PR (95% CI)	*p*-Value	Adjusted PR (95% CI)	*p*-Value†
Age (years)**						
<24	62 (71.3)	25 (28.7)	1.05 (0.96–1.17)	0.29	1.06 (0.97–1.16)	0.18
≥24	102 (53.4)	89 (46.6)	1.03 (1.01–1.05)	**0**.**001**	1.03 (1.01–1.05)	0.01
**Marital status**						
Not married/cohabiting	43 (66.2)	22 (33.8)	0.78 (0.57–1.07)	0.12	1.08 (0.79–1.49)	0.62
Married/cohabiting	119 (56.7)	91 (43.3)	Ref		Ref	
**Marriage type (N = 204)**						
Polygamous	14 (37.8)	23 (62.2)	1.62 (1.09–2.41)	**0**.**02**		
Monogamous	103 (61.7)	64 (38.3)	Ref			
**Partner HIV status**						
Positive	20 (32.8)	41 (67.2)	1.75 (1.14–2.67)	**0**.**01**	1.54 (0.98–2.44)	**0**.**001**
Unknown	104 (68.9)	47 (31.1)	0.81 (0.49–1.34)	0.41	0.79 (0.49–1.29)	
Negative	40 (61.5)	25 (38.5)	Ref		Ref	
**Contraceptive type**						
OCP	11 (64.7)	6 (35.3)	1.03 (0.53–2.00)	0.93		
Condoms only	3 (50.0)	3 (50.0)	1.46 (0.64–3.34)	0.37		
Injectable/implants/IUD	121 (65.8)	63 (34.2)	Ref			
**Condom-less sex**						
Yes	157 (59.5)	107 (40.5)	0.81 (0.48–1.37)	0.43		
No	7 (50.0)	7 (50.0)	ref			
**Transactional sex**						
Yes	4 (33.3)	8 (66.7)	1.67 (1.10–2.54)	**0**.**02**		
No	160 (60.2)	106 (39.9)	Ref			
**STI diagnosis**						
Yes	6 (66.7)	3 (33.3)	0.81 (0.32–2.07)	0.66		
No	158 (58.7)	111 (41.3)	Ref			
**Forced to have sex**						
Yes	7 (50.0)	7 (50.0)	1.23 (0.72–2.11)	0.44		
No	157 (59.5)	107 (40.5)	Ref			
**Experienced IPV**						
Yes	13 (50.0)	13 (50.0)	1.25 (0.86–1.80)	0.24		
No	151 (59.9)	101 (40.1)	Ref			

Bolding denotes statistical significance.

*Row percent.

**†**Adjusted *p*-value for overall effects; for age, this *p*-value is for slope within the respective age group.

**Linear spline with one knot at 24 years.

**Abbreviations:** IPV, intimate partner violence; IUD, intrauterine device; OCP, oral contraception pill; PR, prevalence ratio; PrEP, preexposure prophylaxis; Ref, ; STI, sexually transmitted infection.

Overall, among women with at least one post-PrEP initiation up visit (*n =* 278), no HIV infection was documented during the project period. Self-assessed adherence among women continuing PrEP at month 1 was reported as good (0–3 doses missed in a month) by 93% of the women, highest among women with HIV-positive male partners (partner HIV status: positive 100%, unknown 90%, negative 88%; *p =* 0.05). Overall, in adjusted analyses, early PrEP continuation (i.e. month 1) was strongly associated with reported male-partner HIV status with higher continuation among women with HIV-positive partners (HIV status: positive 67%, negative 38%, unknown 31%; *p* for overall effect = 0.001). Similarly, a higher proportion of women ≥24 years old continued PrEP 1 month post initiation compared with women <24 years old (47% versus 29%; *p =* 0.002). For women ≥24 years old, the likelihood of continuing PrEP at 1 month post initiation increased by approximately 3% for each additional year of a woman’s age (adjusted PR: 1.03; 95% CI: 1.01–1.05; *p =* 0.01). In contrast, for women <24 years old, the likelihood of continuing PrEP for each additional year of a woman’s age was high in magnitude (approximately 6%) but statistically non-significant (adjusted PR: 1.06; 95% CI: 0.97–1.16; *p =* 0.18). Overall, a pattern similar to 1-month continuation was observed for continuation with PrEP use at 3 and 6 months post initiation: 68 of 278 (24%) and 29 of 192 (15%), respectively. For covariates only assessed at the unadjusted level, women in polygamous marriage (*n =* 37; 62% versus 38% in monogamous married; *p =* 0.02) and those who engaged in transactional sex practices (67% versus 40% of those not engaged in transactional sex; *p =* 0.02) were more likely to continue PrEP, but these differences did not persist in the multivariable assessment. There were no discernable statistical differences in early PrEP continuation based on reported history of condom use, STI diagnoses, and IPV or GBV. For continuation at month 3 and 6, only knowledge of male-partner HIV status was statistically significantly associated with continuation of PrEP use in adjusted analyses. Among women who discontinued PrEP (*n =* 123), the most frequently reported reasons for discontinuation were low perceived risk of acquiring HIV (25%), finding out that their male partner was HIV negative (24%), experiencing side effects (20%), and pill burden (17%); few women reported discontinuing PrEP due to fear of IPV (7%).

## Discussion

In this real-world PrEP implementation program, integration of universal screening and counseling for PrEP in FP clinics resulted in PrEP uptake of 22% among HIV-uninfected women, overall, and 16% in AGYW from the general population. Women who initiated PrEP frequently had self-reported behavioral risks for HIV, and more than 40% continued PrEP use beyond 1 month, a continuation rate higher than recently reported from other programs targeting young women within the region [16,17].

FP clinics offer an opportunity for integration of a full complement of sexual and reproductive health services, including PrEP provision and management of STIs, particularly because risk behaviors for unintended pregnancy are similar to those for HIV and STIs and interest in prevention may also extend from pregnancy to HIV/STIs. A recent large clinical trial of contraceptive use and HIV acquisition (ECHO Study) emphasized that HIV risk is high for FP clinic attendees and called for integration of HIV prevention into FP settings [18]. To our knowledge, ours is the first evidence of real-world programmatic delivery of PrEP integrated in routine FP clinics in high–HIV-prevalence settings.

Awareness of PrEP and the individuals’ perceived risk for HIV are important drivers of PrEP initiation and continuation. We found that older women were more likely to perceive or self-assess to be at risk for HIV than AGYW. Women who reported an HIV-positive male partner or those who self-assessed to be at risk of acquiring HIV frequently initiated and continued PrEP. Notably, a substantial proportion of women had partners of unknown HIV status. Many of these women still felt they needed to consult their male partners before they could consider PrEP. Similar to contraception, women have diverse preferences for HIV prevention and need to be empowered to make informed decisions as they strive to achieve their sexual and reproductive health goals while mitigating risks for HIV acquisition. As PrEP comes to scaled implementation, in addition to efforts to create demand, equal priority should be placed on implementation strategies that support women to better evaluate and understand their own risk for HIV, especially AGYW. Such strategies may include distribution of HIV self-test kits to women to efficiently promote and reach male partners for HIV testing [19,20], investing in strategies to increase community PrEP awareness to normalize and minimize stigma for pill taking for HIV prevention in communities where women live, and accelerating delivery of proven HIV prevention options to satisfy the diverse preferences of women and their partners. As new PrEP technologies emerge, including different delivery options such as the dapivirine ring or combined FP and PrEP options, our work will provide informative and important first steps for building robust and integrated FP and HIV prevention systems, including PrEP provision to women at substantial HIV risk in this region.

As expected, a majority of women screened who subsequently initiated PrEP were either using injectable or long-acting reversible contraception methods (i.e., implant or IUD), and pill burden was a common reason for declining PrEP. We also found that younger women used OCP less frequently than older women and that those using OCPs were more likely to initiate PrEP than women using injectable or long-acting reversible contraception methods, possibly because they may have already navigated personal barriers for taking oral medications. Taken together, these findings are important for guiding new directions for PrEP formulation and delivery that respond to the needs of women for whom a daily pill may not be a viable prevention option.

In Africa, PrEP is being added to an already burdened health infrastructure. The ability to build sustainable PrEP programs necessitates making PrEP provision cost-effective and efficient. FP clinics are uniquely important platforms to efficiently reach at-risk women who may benefit from PrEP given that similar factors predispose women to unintended pregnancies and susceptibility for HIV acquisition. Although FP visits are also extremely busy, efficient systems for HIV prevention including PrEP provision can be built into existing routine services. Such implementation strategies may include less frequent PrEP visits and expanding the pool of providers who might be able to screen and provide PrEP beyond the few clinicians and nurses (e.g., training and empowering HIV testing counselors and community health workers or peer educators). These approaches have already been successfully implemented to expand access to injectable and implants contraceptive methods in FP clinics in many African countries using community health workers [21–24]. Similar approaches, commonly described as differentiated care services, are currently being promoted for stable virally suppressed HIV-infected persons in many HIV treatment programs in Africa [25,26].

This work has limitations. First, as for any implementation program, data were collected on standard MOH clinical encounter forms, and thus assessment for correlates of uptake and continuation are limited to covariates included on that standard tool. Second, we assumed that women agreeing to initiate and continue PrEP represented acceptability, but we did not explicitly conduct qualitative interviews with women for insight into young women’s choices, behaviors, beliefs, acceptability, experiences, and priorities as it relates to PrEP. However, an ongoing sister qualitative project that will include women and nurses who participated in this program will provide this contextual information. Third, in the absence of a clear comparator or randomized design coupled with the limited number of measured covariates imposed by the program data, the robust effectiveness of a PrEP-dedicated nurse-led implementation strategy could not be definitively ascertained. Despite these limitations, this work was executed with high rigor consistent with the implementation nature of the program and provides novel evidence for advancing HIV prevention for at-risk adolescents and young women.

In conclusion, integration of universal screening for HIV behavior risk factors and counseling for PrEP in routine FP clinics in Kenya was feasible and resulted in reasonable uptake and continuation in a general population of women including AGYW accessing FP services, which is comparable to continuation of PrEP use observed in general populations in other settings. The enthusiasm for PrEP and evidence demonstrated from this work will set the stage for next steps for full-scale PrEP delivery in FP clinics not only in Kenya but in other settings in Africa. Importantly, this work will lay the foundation for delivery of the next-generation women-controlled PrEP formulations to at-risk young women in this setting.

## Supporting information

S1 Checklist STROBE checklist. STROBE, Strengthening the Reporting of Observational Studies in Epidemiology.
(DOCX)

S1 File Project protocol. (DOCX)

S2 File PrEP risk assessment screening tool. PrEP, preexposure prophylaxis.
(DOCX)

S1 Data Data underlying the manuscript. (CSV)

## Abbreviations

AGYW: adolescent girls and young women
DREAMS: Determined Resilient Empowered AIDS-free Mentored and Safe women
FP: family planning
GBV: gender-based violence
IPV: intimate partner violence
IQR: interquartile range
IUD: intrauterine device
MCH: maternal and child health
MOH: Ministry of Health
NASCOP: National AIDS and STI Control Programme
OCP: oral contraception pill
PEPFAR: President's Emergency Plan For AIDS Relief
PR: prevalence ratio
PrEP: preexposure prophylaxis
PrIYA: PrEP Implementation in Young Women and Adolescents
RAST: risk assessment screening tool
REDCap: Research Electronic Data Capture
STI: sexually transmitted infection
STROBE: Strengthening of the Reporting of Observational Studies in Epidemiology
