# Supplementary material for: Integrating preexposure prophylaxis delivery in routine family planning clinics: A feasibility programmatic evaluation in Kenya
Source: PLoS Med. 2019 Sep 3;16(9):e1002885. doi: 10.1371/journal.pmed.1002885 (PMC6719826; doi:10.1371/journal.pmed.1002885)
Supplement: S1 File — (DOCX) [file pmed.1002885.s002.docx]

**PROTOCOL AND IMPLEMENTATION PLAN**

**Pre-exposure prophylaxis implementation in young women and adolescents in**

**Kenya**

**DREAMS Innovation Challenge—PrIYA**

Version 4.0

November 13, 2017

Table of Contents

[1) TITLE: 4](#_Toc477168897)

[2) INVESTIGATORS (Roles and responsibilities): 4](#_Toc477168898)

[3) COLLABORATING INSTITUTIONS 7](#_Toc477168899)

[4) FUNDING AGENCY 7](#_Toc477168900)

[5) EXECUTIVE SUMMARY 8](#_Toc477168901)

[6) BACKGROUND AND RATIONALE 10](#_Toc477168902)

[7) IMPLEMENTATION APPROACH 15](#_Toc477168903)

[OBJECTIVES 15](#_Toc477168904)

[BROAD OBJECTIVE 15](#_Toc477168905)

[SPECIFIC OBJECTIVES 15](#_Toc477168906)

[DESIGN 16](#_Toc477168907)

[SETTING 16](#_Toc477168908)

[PROGRAM INCLUSION/EXCLUSION 16](#_Toc477168909)

[RECRUITMENT PROCEDURES 17](#_Toc477168910)

[PROGRAM PROCEDURES/ACTIVITIES 17](#_Toc477168911)

[LABORATORY METHODS 21](#_Toc477168912)

[RESEARCH COMPONENT 21](#_Toc477168913)

[DATA 22](#_Toc477168914)

[HEALTH ECONOMICS 22](#_Toc477168915)

[QUALITY ASSURANCE PROCEDURES 23](#_Toc477168916)

[DISSEMINATION 23](#_Toc477168917)

[8) HUMAN SUBJECTS CONSIDERATIONS 23](#_Toc477168918)

[RISKS 24](#_Toc477168919)

[BENEFITS 24](#_Toc477168920)

[TREATMENT FOR INJURY 24](#_Toc477168921)

[INFORMED CONSENT 24](#_Toc477168922)

[9) PROGRAM RECORDS 26](#_Toc477168923)

[DATA OWNERSHIP 26](#_Toc477168924)

[DATA RELEASE/SHARING POLICY 26](#_Toc477168925)

[CONFIDENTIALITY 26](#_Toc477168926)

[10) LIMITATIONS 26](#_Toc477168927)

[11) TIMELINE 26](#_Toc477168928)

[12) EXPECTED APPLICATION OF RESULTS 27](#_Toc477168929)

[14) REFERENCES 28](#_Toc477168930)

**LIST OF ABBREVIATIONS**

**AGYW** Adolescent Girls and Young Women

**DBS** Dried Blood Spots

**DREAMS** DREAMS Innovation Challenge Program

**ERC** Ethical Review Committee

**FP** Family Planning

**GBV** Gender-based Violence

**HIV** Human Immunodeficiency Virus

**IEM** Information Education Materials

**IPV** Intimate Partner Violence

**IRB** Institutional Review Board

**MCH** Maternal Child Health

**MOH** Ministry of Health

**NASCOP** National AIDS and STI Control Program

**POC** Point of Care

**PrIYA** PrEP Implementation for Young Women and Adolescents

**PrEP** Pre-Exposure Prophylaxis

**SMS** Short Message Service

1. TITLE: Pre-exposure prophylaxis implementation in young women and adolescents in Kenya: DREAMS Innovation Challenge--PrIYA

# INVESTIGATORS (Roles and responsibilities):

Grace John-Stewart, MD, PhD (MULTIPLE PRINCIPAL INVESTIGATOR)

(Responsible for the overall program development, oversight, and science of Maternal and Child Health components of the program)

Professor

Departments of Global Health, Medicine, Epidemiology and Pediatrics, University of Washington

Harborview Medical Center, 325 Ninth Ave., Box 359909, Seattle, WA 98104

Tel: +1-206-543-4278

Fax: +1-206-543-4818

Email: [gjohn@uw.edu](mailto:gjohn@uw.edu)

Jared Baeten, MD, PhD (MULTIPLE PRINCIPAL INVESTIGATOR)

(Responsible for the overall program development and oversight and science of PrEP components of the program)

Professor, Vice Chair

Departments of Global Health, Medicine, and Epidemiology, University of Washington

Harborview Medical Center, 325 Ninth Ave., Box 359927, Seattle, WA 98104

Tel: +1-206-520-3808

Fax: +1-206-520-3831

Email: [jbaeten@uw.edu](mailto:gjohn@uw.edu)

John Kinuthia, MBChB, MMed, MPH (SITE PRINCIPAL INVESTIGATOR)

(Responsible for the development and overall program oversight and science and overseeing all implementing team members in Kenya)

Head, Department of Research & Programs, Kenyatta National Hospital

PO Box [20723-00202](tel:20723-00202), Nairobi, Kenya

Tel: +254 0722 799-052

Email: [kinuthia@uw.edu](mailto:kinuthia@uw.edu)

Kenneth Mugwanya, MBChB, MS, PhD (PROGRAM DIRECTOR)

(Responsible for protocol, data and analysis plan, science of PrEP components of the program, and program coordination)

International Clinical Research Center, Department of Global Health, University of Washington

Harborview Medical Center, 325 Ninth Ave., Box 359927, Seattle, WA 98104

Tel: +1- 206-520-3806

Email: mugwanya@uw.edu

Ruanne Barnabas, MD, DPhil (CO-INVESTIGATOR, Program Health Economist)

(Responsible for protocol, data and analysis plan, and evaluation of the health economics)

Assistant Professor

Departments of Global Health, Allergy and Infectious Diseases, University of Washington

Harborview Medical Center, 325 Ninth Ave., Box 359927, Seattle, WA 98104

Tel: +1-206-520-3813

Fax: +1-206-520-3831

Email: [rbarnaba@uw.edu](mailto:rbarnaba@uw.edu)

Carol Levin, PhD (CO-INVESTIGATOR, Program Health Economics)

(Responsible for protocol, data and analysis plan, and evaluation of the health economics)

Senior Research Scientist

Departments of Global Health, University of Washington

Tel: +1-206-744-3694

Email: clevin@uw.edu

Gabrielle O’Malley, PhD (CO-INVESTIGATOR, Monitoring and Evaluation lead)

(Responsible for protocol, data and analysis plan, and evaluation of the qualitative)

Associate Professor

Department of Global Health, University of Washington

International Training and Education Center for Health (I-TECH), 902 Boren Ave, Suite 1100, Box 359932, Seattle, WA 98104

Tel: +1-206-685-0775

Email: [gomalley@u.washington.edu](mailto:gomalley@u.washington.edu)

Jennifer Unger, MD, MPH (CO-INVESTIGATOR, SMS Mobile WACh program)

(Responsible for protocol, development and implementation of the SMS adherence program)

Assistant Professor

Department of Global Health, University of Washington

Harborview Medical Center, 325 Ninth Ave., Box 356460, Seattle, WA 98104

Tel: +1-206-520-3869

Email: junger@u.washington.edu

Alison Drake, PhD (CO-INVESTIGATOR, SMS Mobile WACh program)

(Responsible for protocol, development and implementation of the SMS adherence program)

Assistant Professor

Department of Global Health, University of Washington

Harborview Medical Center, 325 Ninth Ave., Box 359909

Tel: 206 543-5847

Email: adrake2@uw.edu

Harsha Thirumurthy, PhD (CO-INVESTIGATOR, Self-Testing Lead)

(Responsible for protocol, data and analysis plan, and evaluation for self-testing components of the study and support to health economics aspects of the study)

Associate Professor of Health Economics

Department of Health Policy and Management, Gillings School of Public Health

University of North Carolina, Chapel Hill

Tel: +1-919- 966-9756

Email: harsha@unc.edu

Kristin Beima-Sofie, PhD (CO-INVESTIGATOR)

(Responsible for protocol, develop and refine intervention, work closely with M&E lead)

Senior Fellow

Department of Global Health, University of Washington

Harborview Medical Center, 325 Ninth Ave., 359909

Tel: +1-206-520-3869

Email: [beimak@uw.edu](mailto:beimak@uw.edu)

Dr. Anne Njoroge, MBChB, MPH (SITE PROGRAM COORDINATOR)

(Responsible for program coordination, ERC communication, protocol development, data collection, and analysis)

Research & Programs,

Kenyatta National Hospital

PO Box [20723-00202](tel:20723-00202), Nairobi, Kenya

Tel: 0722493595

Email: anjoroge@uw.edu

Martin Sirengo, MBChB, MMed (CO-INVESTIGATOR, NASCOP)

(Responsible for guiding program development and implementation to align with NASCOP activities)

National AIDS and STI Control Program (NASCOP)

Ministry of Health, Nairobi Kenya

P.O. Box 19361-00202

Nairobi, Kenya

Tel: +254 20 2729502

Email: sirengomartins@gmail.com

Felix Abuna Otieno, BA (SITE PROGRAM ASS. COORDINATOR)

(Responsible for lab and site protocol development, ERC Communication, data analysis and staff oversight)

Obstetrics and Gynecology

University of Nairobi

Nairobi, Kenya

Tel: 0721230652

Email: [fabuna@yahoo.com](mailto:fabuna@yahoo.com)

Julia Dettinger, MPH (PROGRAM COORDINATOR)

(Responsible for program coordination, IRB communication, protocol development, data collection, and analysis)

Department of Global Health, University of Washington

Harborview Medical Center, 325 Ninth Ave., Box 359931, Seattle, WA 98104

Tel: +1-206-221-1041

Fax: +1-206-744-3693

Email: [jcdettin@uw.edu](mailto:jcdettin@uw.edu)

Tina Schuh, MPH (PROGRAM COORDINATOR)

(Responsible for program coordination, IRB communication, protocol development, data collection, and analysis)

International Clinical Research Center, Department of Global Health, University of Washington

Harborview Medical Center, 325 Ninth Ave., Box 359931, Seattle, WA 98104

Tel: +1-206-520-3869

Fax: +1-206-744-3693

Email: [tschuh@uw.edu](mailto:tschuh@uw.edu)

# COLLABORATING INSTITUTIONS

Kenyatta National Hospital, University of Colorado, and University of Washington.

# FUNDING AGENCY

**Funding type:** Grant

**Name of Funding agency:** The U.S. President’s Emergency Plan for AIDS Relief

**Principal Investigator on Proposal:** Grace John-Stewart, Jared Baeten and John Kinuthia

**Proposal Identification Number:** S-LMAQM-16-CA-1103

**Title of Proposal:** Pre-exposure prophylaxis implementation in young women and adolescents in Kenya: DREAMS Innovation Challenge--PrIYA

**Dates: 10/1/2016 – 09/30/2018**

# EXECUTIVE SUMMARY

In Africa, women are disproportionately affected by HIV because of biological, cultural, and structure reasons. Moreover, pregnancy and postpartum periods are associated with >2 fold increased HIV acquisition risk because of infrequent condom use, unknown partner’s HIV status, and biologic changes or changes in their male partner’s sexual partnerships that increase susceptibility. Oral pre-exposure antiretroviral prophylaxis (PrEP) is a highly effective and an attractive HIV prevention strategy for women because it does not require negotiation for safe sex or interfere with pregnancy or breastfeeding. It provides dual protection of her child, as transmission to infants is enhanced when women have acute HIV infection during breastfeeding. However, it is important to ensure PrEP reaches women at risk for acquiring HIV while avoiding unnecessary PrEP use during low risk periods.

The World Health Organization recommends PrEP for all persons at substantial risk of HIV acquisition. The Kenya Ministry of Health has developed an ambitious HIV Prevention Revolution Road Map 2030, including provision of PrEP for persons with a substantial HIV risk. In July 2016, the Ministry of Health in Kenya released “Guidelines on use of Antiretroviral Drugs for Treating and Preventing HIV infections in Kenya” which recommend oral PrEP for HIV uninfected persons with substantial ongoing risk of HIV infection. However, delivery approaches that will catalyze scale-up of PrEP have not yet been implemented. MCH and some FP clinics have adapted to include HIV testing and other prevention services and these clinics are highly attended, thus offering an existing platform for incorporating clinical prevention services including PrEP delivery for young women that is less stigmatizing than at HIV-specific clinics. In collaboration with NASCOP, this program will integrate and scale-up PrEP counselling and delivery for young women and adolescents in 21 MCH and FP clinics in Kisumu County, Kenya. We will conduct quality improvement evaluation to identify suitable and efficient models of PrEP counselling, implementation barriers and solutions, characterize costs, and provide best practices for further scale-up.

**Design**: An implementation program to scale-up PrEP counselling and delivery to young women adolescents at high risk for HIV in public health sector MCH and FP clinics.

**Population:** Heterosexual HIV uninfected women 15-45 years attending MCH and FP clinics. We will approach 16000 women overall and we anticipate that 50 % (8000) of the clients will be women aged 15-24 years, a priority group for HIV prevention.

**Objective 1: Deliver PrEP to young women and adolescents at high risk for HIV through highly accessed public sector MCH and FP clinics and evaluate efficient models of delivery**. In collaboration with NASCOP, we will introduce PrEP implementation in 24 highly accessed public sector MCH and FP clinics according to Kenya national guidelines. Clinics will implement either universal consideration for PrEP (model 1; 12clinics) or male partner self-testing guided PrEP consideration (model 2; 12 clinics). The most efficient and cost-effective model will be recommended to the MoH for scale-up in other clinics in Kenya.

**Objective 2: Build capacity and streamline HIV prevention services in public sector MCH and FP clinics that include PrEP and encourage male partner HIV testing.** In collaboration with NASCOP and the County government, we will provide the necessary foundation to enhance HIV prevention systems to scale-up PrEP implementation in MCH and FP clinics.

**Objective 3: Assess cost, and cost-effectiveness of delivering PrEP in public sector MCH and FP clinics to help decision makers define priorities and allocate resources.**

**Approach:** Data from the program will be used to conduct cost-effectiveness analyses to compare costs of offering and delivering PrEP in MCH and FP clinics

# BACKGROUND AND RATIONALE

**Importance of the Problem**

More than two million persons are infected with HIV-1 each year, the majority among women in Africa [1]. In Kenya, 21% of all new adult HIV infections occur in young women 15-24 years. Population scale-up of effective female-controlled HIV prevention interventions remains a global priority. Antiretroviral pre-exposure prophylaxis (PrEP) is a highly effective and recommended HIV prevention option for women that does not require negotiation for safer sex. Thus, successful delivery of PrEP for HIV-1 prevention, at scale and tailored to the epidemiologic profile of priority populations, has the potential to reverse the global epidemic [[2](#_ENREF_2), [3](#_ENREF_3)].

**Adolescents and Young Women are a Priority Population for Prevention Services**

In Africa, adolescent girls and young women accounted for 25% of new HIV infections among adults in 2015, and 56% of all new HIV infections among adults occurred in women. Moreover, adolescent girls in Africa have a 3-fold higher HIV prevalence than their male counterparts (UNAIDS, 2013). High HIV incidence among young women in Africa highlights the need for urgent scale-up of proven female-controlled HIV prevention strategies. Social, structural, and economic disparities perpetuate vulnerabilities of women. The lower social and economic power of women makes it difficult for them to negotiate safe sex. In stable partnerships, condom use is low and women are often unaware of their partner’s HIV status [4]. Young women may partner with older men for financial support or based on cultural norms. Lack of money, time, and awareness make it difficult for women to seek VCT, condoms, or PrEP.

**High risk of HIV Acquisition in Pregnancy and During Breastfeeding**

Women have additional reasons for heightened HIV risk during periods when they attend MCH and FP clinics. Women have biologic changes in pregnancy and mucosal disruption at delivery, and male partners may have other sexual partners and bring HIV back to women following periods of perinatal abstinence. In addition to the biological changes that put women at higher risk during pregnancy and postpartum, cofactors for unprotected sex during the postpartum period were mostly similar to those factors during pregnancy (Kinuthia et al. 2015). Condom use, though effective, remains low in many sub-Saharan African settings among pregnant and postpartum women. Lack of condom use in addition to high rates of unknown partner HIV status and polygamy highlights the need for female-controlled HIV prevention interventions (Kinuthia et al., 2015).

In a meta- analysis of 19 studies including 22,803 person-years, HIV incidence in pregnant/postpartum women was 3.8 per 100 p-years, 4.7/100 p-years in pregnancy and 2.9/100 p-years postpartum [5]. In Kenya specifically, incidence in pregnant and postpartum women was 6.8 per 100 p-years [5]. These HIV incidence estimates are particularly high given that the estimation was among all women (not limited to those with seropositive partners) and occurred despite decreased sexual activity in late pregnancy and early postpartum. These HIV incidence estimates in pregnancy and postpartum women are as high or higher than many ‘high risk’ groups, including sex-workers. Over 40% of new infant HIV infections worldwide are estimated to be due to maternal HIV acquisition in pregnancy and postpartum. Women have persistent risk of acquiring HIV in pregnancy and postpartum and often do not perceive themselves to be at risk. This analysis highlights the urgent need for scale-up of to effective HIV prevention to young women including in the pregnancy and postpartum period.

Integrating FP and MCH services with HIV services is a recognized a key strategy to meet the 2015 MDG [6]. By adding PrEP counselling and dispensing to MCH and FP clinics it is possible to expand access to an already established at risk group of pregnant, post-natal and women accessing FP services. The combined need for HIV protection during pregnancy and when seeking contraception, both widely accessed services, and the efficiency gained from leveraging existing MCH and FP platform, make this solution a powerful approach for HIV prevention in young women. Integrating family planning services, care at MCH clinics, and HIV counselling and testing reduces the chance of missing women as opposed to when the services are separate.

In Kenya, 95% of pregnant women aged 15-24 receive care at MCH clinics and 35% of sexually active women aged 15-24 receive family planning services at public facilities [7]. Using the existing health systems circumvents the structural barriers that women often face including the lack of time, cost, and potential stigma of visiting a facility solely for HIV prevention. FP and MCH clinics provide broad coverage for women in their reproductive years.

**PrEP Prevents HIV Acquisition in Women: Implementation Science is Needed to Translate Scientific Advances into Public Health Impact**

PrEP is an attractive strategy for HIV prevention however, it is important to ensure PrEP reaches women who are at risk for acquiring HIV. Targeting PrEP to women at the greatest risk of HIV may maximize benefits, minimize potential risks, and optimize cost-effectiveness.

**Table 1 TDF-based effectiveness against HIV acquisition. Adapted from Kerry et al Curr Opin HIV/AIDS 2016**

|  | Oral PrEP | Tenofovir level in random plasma samples (%) | Overall efficacy | Female sub-group efficacy | efficacy in women <30 yrs |
| --- | --- | --- | --- | --- | --- |
| Baeten et al  Donell et al | FTC-TDF  TDF | 81  83 | 75%  67% | 66%  71% | 72%  77% |
| Thigpen et al | FTC-TDF | 79 | 62% | 49% | 77% |
| Choopanya et al | TDF | 67 | 49% | 79% | 75% |
| Van Damme et al | TDF | 24 | 6% |  |  |
| Marrazo et al | FTC-TDF  TDF | 29  30 | -4%  -49% |  |  |

Our landmark Partner PrEP study demonstrated that oral PrEP is equally protective in women and men when taken with sufficient adherence including in women at high HIV risk including those <30 years [8-10] , women using contraception, and in couples with high-risk characteristics (e.g., those practicing unprotected sex, couples in which the HIV-1 infected partner had a high plasma viral load, those with sexually transmitted infections) (Table 1). These data are further collaborated by sub-group analyses of women in TDF2 Study in African heterosexual men and women and the Bangkok Tenofovir study have demonstrated protection in women[11,12].

Although two trials in African heterosexual women showed disparate results, there is substantial objective evidence indicating that these two studies were limited by low adherence to study medication. These Challenges can be minimized by offering women PrEP counselling and dispensing at the MCH and FP clinics that women are already visiting. Logistically women can attend their MCH and FP appointments without the extra pressures and stigmas of attending HIV clinics. Emerging evidence from initial PrEP implementation and demonstration studies indicate that adherence is high in open label “real world settings when women have knowledge of HIV prevention benefits of PrEP [13-15].

**Programmatic Delivery Requires Implementation Evaluations**

**Figure 1. Steps to program delivery (AVAC)**

Proof of efficacy in clinical trials is only the first step towards public health impact. Many effective interventions in health are not implemented because of inadequate knowledge how to deliver. For novel HIV prevention interventions, like PrEP for prevention, a pathway for this transition to implementation has been described ([16] and **Figure 1**): clinical trials are followed by open label extensions, which provide first access to the product in the trial population, then demonstration projects, which “road test” the intervention among individuals who had not been in the clinical trials and using strategies to mimic real-world delivery, and finally product introduction, which leads the intervention to scale while integrating into existing health systems. Along this pathway, implementation science can guide optimization. We have followed this pathway in moving PrEP from a clinical trial (the Partners PrEP Study) to an open-label extension (the Partners PrEP Study Open-Label Extension) to a demonstration (the Partners Demonstration Project). Bringing this successful intervention to scale requires an implementation science evaluation in public health clinics.

**The Government of Kenya’s Plan for Evidence-Based HIV Prevention**

The Kenya Ministry of Health developed the HIV Prevention Revolution Road Map: Count Down to 2030, a national plan to drive new HIV infections towards zero (<http://hivhealthclearinghouse.unesco.org/sites/default/files/resources/kenya_hiv_prevention_revolution_road_map.pdf>). The approach is based on key concepts in combination prevention: prioritization of sub-populations (young women at HIV risk, HIV serodiscordant couples, sex workers, men who have sex with men) and delivery of evidence-based combination prevention; these are priorities for prevention for WHO and PEPFAR as well. The 2012 Kenya AIDS Indicator Survey, a population-based national survey, estimated that 56% of new adult infections (≥15 years) occurred in women. In 2012, 1.2 million persons in Kenya were living with HIV and >100,000 became infected, the fourth largest epidemic in the world. Our proposed work aligns with the Kenya HIV Prevention Revolution Road Map strategy for young women (**Table 2**). This visionary document calls out key gaps in research and delivery, including calls for scale-up evaluations. In July 2016, the Ministry of Health in Kenya released “Guidelines on use of Antiretroviral Drugs for Treating and Preventing HIV infections in Kenya” which recommend initiation of ART for all HIV infected persons irrespective of CD4 count and oral PrEP for HIV uninfected persons with substantial ongoing risk of HIV infection, including young women [17].

**Table 2. Combination prevention for young women at risk 15-24 yrs, from the Kenya Prevention Revolution Road Map**

| **Priority Population** | **Biomedical Interventions** | | **Behavioural** | **Structural** |
| --- | --- | --- | --- | --- |
|  | **Community Settings** | **Facility Settings** |  |  |
| **Young women at risk 15-24 years** | HTC and STI, HPV screening and education + 100% condom, EC | FP, PrEP, PEP, EMTCT | Healthy choices | Keep girls in school  Cash transfer for out of school if stay negative  GBV program |

HTC= HIV Testing and Counseling; STI= Sexually Transmitted Infection; FP = Family planning; PrEP=pre-exposure prophylaxis; PEP= post exposure prophylaxis; GBV= Gender Based Violence; EMTCT = elimination of mother-to-child transmission

**Evidence for Antiretroviral Pre-exposure Prophylaxis for HIV Prevention in Kenya**

Since 2004, our collaborative team has conducted high-impact, collaborative HIV prevention studies involving >4500 African women (**Table 3**); all received risk reduction counselling (for the individual and couple), condoms, and sexually transmitted infection treatment. Study conduct was outstanding (e.g., retention >95%). We have used the wealth of data from these studies to build the evidence base for optimized HIV prevention for African HIV serodiscordant couples, particularly for ART and PrEP. Our results have been an important part of the development of Kenya, FDA, CDC, and WHO PrEP guidance [18, 19].

**Table 3. Experience with multisite prospective studies of HIV-1 serodiscordant couples**

|  | **Partners in Prevention HSV/HIV Transmission Study** | **Couples Observational Study** | **Partners PrEP Study & Open-Label Extension** | **Partners Demonstration Project** |
| --- | --- | --- | --- | --- |
| Sample size | 3,408 couples | 485 couples | 4,747 couples | 1,013 couples |
| Location | Botswana, Kenya, Rwanda, South Africa, Tanzania, Uganda, Zambia | Uganda,  South Africa | Kenya, Uganda | Kenya, Uganda |
| Follow-up | Monthly for 12-24 months | Quarterly for 12 months | Monthly for 24-48 months | Quarterly for 24 months |
| Primary aim | RCT of HSV-2 suppression | Immune correlates of HIV-1 | RCT of PrEP | PrEP demonstration project |
| Timeline | 2004-2008 | 2007-2010 | 2008-2013 | 2012-2016 |

**Male Partner Self-testing**

Using only a behavior assessment may not identify all women at risk of HIV. Offering male partner self-testing is a key strategy that has been successfully used to identify previously unknown HIV-positive partners of women in Western Kenya visiting MCH and FP clinics. By offering HIV self-tests HIV diagnosis and treatment can be fast-tracked for men, who are often under-represented in HIV clinics. Male partners have also proven to be a challenging group to access with provider-initiated strategies. Our team recently piloted a novel study of male partner HIV testing through distribution of self-tests to HIV-negative women in MCH and FP clinics in Western Kenya [20]. The study demonstrated high uptake and acceptability of partner-testing when multiple self-tests are distributed to women in MCH and FP clinics and the strategy was found to be safe for women who participated.

**Safety of PrEP by HIV-uninfected Pregnant and Breastfeeding Women**

PrEP is safe and effective for HIV protection during pregnancy and lactation. In the Partners PrEP trial, women who became pregnant on PrEP had no evidence of adverse infant outcomes or long-term growth. Pregnancy outcomes were similar between placebo and PrEP arms among 431 women who became pregnant in this trial and so were infant growth outcomes (weight, height, and head circumference) among 167 infants with serial growth assessment. While safety data are generally reassuring, there is need for continued accrual of data on growth and pregnancy outcomes in PrEP research studies and demonstration projects. Breastfeeding is not a contraindication for maternal PrEP as minimal drug is likely to pass to infants. Our data (PrEP in breastfeeding study) has shown that very low concentrations of tenofovir were detectable in breast milk and tenofovir was undetectable in 94% plasma of infants breastfed by women using PrEP for HIV prevention. The data also strongly suggests that TDF and TDF/FTC can safely be given to breastfeeding women without putting their infants at risk of adverse effect [21-23]. These results make PrEP a safe option for HIV prevention for women (and their babies) utilizing both MCH and FP clinics.

**FP and PrEP**

Women who can utilize PrEP as an HIV prevention strategy and the use of hormonal contraception can utilize dual protection from HIV and pregnancy. It has been established that PrEP has no adverse impact on hormonal contraceptive effectiveness for pregnancy prevention. In addition, hormonal contraception does not affect PrEP effectiveness [24]. This data makes PrEP a safe option for women at risk for HIV who are utilizing FP clinics and who wish to abstain from pregnancy.

**Summary**

PrEP is safe and effective for HIV prevention when taken with sufficient adherence including in young women and women using contraception. The next step is to scale-up delivery in the public health facilities to make delivery of this highly effective prevention intervention achievable in real-world settings. The proposed work will build on and extend beyond our prior work, building capacity throughout the program period and ending with a public health intervention at scale.

# IMPLEMENTATION APPROACH

As part of the Kenya national PrEP scale-up program, we will introduce PrEP counselling and delivery in highly accessed MCH and FP clinics according to Kenyan national guidelines. NASCOP PrEP delivery tools will be adapted and operationalized in MCH and FP clinics including the PrEP booklet (clinical patient information card used as a clinical monitoring tool in Kenya). The program will support participating clinics’ readiness to deliver PrEP as part of a combination HIV prevention package. Program evaluation will characterize implementation process, efficiency, and costs in order to identify what is working and what is not working so that services can be improved as the program scales-up. We will characterize models of counselling and delivering PrEP in MCH and FP clinics which efficiently reach and cover women during periods of substantial HIV risk.

## OBJECTIVES

### BROAD OBJECTIVE

To integrate PrEP counselling and delivery as part of routine HIV prevention services in MCH and FP clinics in Kenya

### SPECIFIC OBJECTIVES

**Objective 1: Deliver PrEP to young women and adolescents at high risk for HIV through highly accessed public sector MCH and FP clinics and evaluate efficient models of delivery.**

**Approach**: We will introduce PrEP counselling and delivery to young women and adolescent at high risk for HIV acquisition in 24highly accessed MCH and FP clinics, and we will characterize the performance of two potential models of PrEP counselling: Universal consideration for PrEP or Male partner self-testing guided PrEP consideration. The program will be evaluated for improvement in an ongoing way and lessons learned will inform scale-up in other clinics in Kenya.

**Objective 2: Build capacity and streamline HIV prevention services in public sector MCH and FP clinics that include delivering PrEP to women at high risk for HIV and encourage male partner HIV testing.**

**Approach**: In collaboration with NASCOP and the County governments, we will provide the necessary foundation to enhance HIV prevention systems to scale-up PrEP implementation in MCH and FP clinics. The program will build capacity in implementing clinics in the following areas; 1) utilization of tools to implement PrEP; 2) male partner self-testing; 3) train staff and provide technical assistance for PrEP counselling and delivery; 4) harmonized systems for tracking women on PrEP.

**Objective 3: Assess cost, and cost-effectiveness of integrating PrEP counselling and delivering PrEP in public sector MCH and FP clinics to help decision makers define priorities and allocate resources.**

**Approach:** We will conduct time and motion studies, define the costs and model the cost-effectiveness of two models of PrEP counselling and delivery (Objective 1) in MCH and FP clinics in terms of HIV infections averted, disability-adjusted life years saved, and incremental cost-effectiveness over standard MCH/FP care.

## DESIGN

This is an implementation program to scale-up PrEP counselling and delivery to young women and adolescents at high risk for HIV in public health sector MCH and FP clinics in Kenya. The program is made up of two distinct but complementary components: 1) a PrEP implementation component that includes procedures for counseling and delivering PrEP to at-risk women as part of routine care and 2) a nested research component to collect dried blood spots to test tenofovir levels in a subset of women using PrEP.

POPULATION

The program will be set up for all HIV uninfected women of reproductive age, 15-45 years old, receiving routine MCH and FP services. We will target to reach (i.e approach and counsel on PrEP) 16,000 clients or approximately 1000 at each participating clinic.

SETTING

The proposed program will be implemented in 24high volume MCH and FP (12 MCH, 12FP) clinics in Kisumu County, Kenya. These are derived from both the public and private sector. Clinics to implement the program were selected based on volume and geography in consultation with the Kisumu County health authorities. The list of proposed clinics is in Table 4.

**Table 4:** Facility and Clinic List:

|  | **Facility** | **Clinic** |
| --- | --- | --- |
| 1. | Chulaimbo County Hospital | MCH/ANC |
| 2. | Muhoroni County Hospital | MCH/ANC |
| 3. | Nightingale Medical Center | MCH/ANC |
| 4. | Nyakach County Hopsital | MCH/ANC |
| 5. | St. Elizabeth Mission Hospital Chiga | Both |
| 6. | Awasi Mission hospital | Both |
| 7. | Koru Mission hospital | Both |
| 8. | Airport Dispensary | FP |
| 9. | Manywanda sub-county hospital | FP |
| 10. | Nyalenda Health Center | FP |
| 11. | Rabour Sub-county hospital | FP |
| 12. | Ahero County Hospital | Both |
| 13. | Kisumu County Hospital | Both |
| 14. | Lumumba sub-county hospital | Both |
| 15. | Migosi sub-county hospital | Both |
| 16. | Maseno Mission Hospital | Both |

## PROGRAM INCLUSION/EXCLUSION

The program is set up to for all HIV negative women 15-45 years receiving MCH/ANC and FP services including pregnant women and breastfeeding women.

## RECRUITMENT PROCEDURES

For this PrEP roll-out program, where the goal is for real-world delivery of PrEP as part of national PrEP scale-up program, formal recruitment will reflect approaches used by MCH and FP clinics in Kenya. Following routine MCH and FP clinic visit schedule, all women entering the clinic for services will be counseled about PrEP as part of standard HIV prevention services in these clinics.

## PROGRAM PROCEDURES/ACTIVITIES

The proposed program is made up of two distinct but complementary components:

1. PREP **PROGRAM IMPLEMENTATION PROCEDURES:** Includes activities that are part of day-to-day HIV preventions services including services for mpersons on PrEP or will support integration of counseling and delivering of PrEP to women in routine MCH and FP services that include abstraction of some data for quality improvement.
2. **RESEARCH PROCEDURES: The research component for the program will include** collection of dried blood spots for drug levels to evaluate adherence to PrEP in the program in a subset of women who choose to use PrEP.

**PrEP IMPLEMENTATION PROCEDURES**

To achieve the program objectives, the following activities will be implemented as part of routine services provided to women attending MCH and FP clinics:

***PrEP implementation in MCH and FP clinics***

We will introduce PrEP counseling and delivery as part of routine HIV prevention services to women of reproductive age 15-45 years receiving routine care in 24 MCH and FP clinics (12 MCH and 12 MCH) in Kisumu County. We will target to approach and counsel 16,000 new clients per clinic. Women with behavior risk characteristics that put them at substantial risk for HIV will receive counselling and will be offered PrEP according to national guidelines. We anticipate that 20% (approximately up to 200 women per clinic) of these women will be eligible and choose to use PrEP as an additional HIV prevention option. PrEP eligibility will be assessed by standard behavior risk assessment criteria developed by NASCOP and these characteristic include: a) Inconsistent or condom use; b) having a sex partner(s) high risk & HIV status is unknown; c) engaging in transactional sex; d) Ongoing Intimate Partner Violence (IPV) and Gender-based Violence (GBV); e) Recent bacterial sexually transmitted infection; f) Recurrent use of post-exposure prophylaxis; g) Recurrent sex under influence of alcohol/recreational drugs; h) Injection drug use with shared needles and/or syringes; and i) In HIV serodicordant partnership where the HIV-infected has either not initiated ART or is not virally suppressed or a couple wants to conceive.

*Models of Counselling and Delivering PrEP in MCH and PC Clinics*

There are no clear delivery approaches to catalyze scale-up of PrEP to young women and adolescent girls in Kenya. We will implement two model of PrEP counseling: **Universal consideration for PrEP** (model 1) or **Male partner self-testing guided PrEP consideration** (model 2). Clinics of comparable size will implement either model 1 or model 2. In the course PrEP implementation, we will characterize the performance of the model to identify which model efficiently best identifies women that might benefit from PrEP.

**In clinics implementing universal consideration for PrEP model,** all women will be counselled and assessed for HIV risk based on standard NASCOP behavior risk; women at substantial risk for HIV acquisition will be counselled and offered PrEP per Kenyan national clinical guidelines for PrEP*.* This approach will be implemented in 12clinics (6MCH, 6FP; ~ 8000 women)

**In clinics implementing male partner self-testing-guided PrEP consideration model,** male partner self-testing will be used to further refine identification of women at high risk for HIV who would benefit from PrEP counselling. The Kenya HIV testing services guidelines support self-testing to act as catalyst towards increasing access to and coverage of HIV testing (<https://archive.org/details/hts_policy_kenya_2015>), and male partner engagement and HIV testing is part of existing MCH and FP services in Kenya.

***Partner self-testing***

The program will enhance the existing systems in MCH and FP clinics to encourage HIV self-testing male partner. Women attending clinics implementing male partner self-testing-guided PrEP consideration (model 2) will be offered the opportunity to deliver saliva HIV self-test kits to their partners. Partner self-testing will be implemented as follows. First, women who believe violence could occur when providing the self-testing kit to their partner will not be offered testing kits and this information but will continue to receive all other HIV services including PrEP. Second, women who are comfortable delivering a self-test to their sexual partner(s) will be given instruction by trained clinic staff on how to use saliva HIV self-test. Women will receive additional counselling about using discretion when determining whether to offer a self-test to sexual partners. Women will be offered two self-tests so that they can take the test at the same time as their partner, if they chose to do so.

In this approach (Model 2), women will be identified as at high risk for HIV acquisition and considered for PrEP through three mechanisms: (1) through standard HIV risk assessment, (2) by declining partner-self test for HIV with a partner of unknown HIV status, or (3) a previously unknown HIV-positive partner identified through self-testing. Women at high risk for HIV who are medically eligible and elect to initiate PrEP will be provided with PrEP. This model will be implemented in 12 clinics (6MCH, 6FP; ~ 8000 women).

Women will be given a helpline phone number to call if they have questions or concerns about the self-test before, during or after accepting the partner self-test. We will utilize the OnetoOne® hotline by LVCT that has been set forth as the NASCOP hotline.. Follow-up information about the partner self-test process will be obtained from women by self-report using a brief standardized tool at their routine follow-up visits at all clinics. . The active follow-up will be conducted using automated SMS messages that will be sent by a third party, specifically mSurvey®. The participant will receive a maximum of 6 SMS and will not be charged for the responses. If a participant does not respond to the SMS, a nurse will call the participant to ask the same questions. The nurse will attempt to reach the participant twice.

Follow-up information will inquire whether the partner received the kits, used the kits, test results, and if there was any harm or negative reactions from the partner regarding the test. As part of the partner self-testing protocol for targeted PrEP administration facilities, participants will be asked to report their male partner’s age and HIV test results, if they feel comfortable doing so. Participants will be asked whether their partner showed them the test results or if their partner simply reported them. Study staff will not collect identifiable information about the partner as part of the partner self-testing activities.

***PrEP Medications***

HIV uninfected women at substantial risk for HIV infection who chose to initiate PrEP and are medically eligible will receive PrEP as part of the Kenya national PrEP scale-up program. PrEP medication and dosing will follow the 2016 Guidelines on Use of Antiretroviral Drugs for Treating and Preventing HIV Infections in Kenya. Oral daily tenofovir disoproxil fumarate/emtricitabine (300mg/200mg) is the approved PrEP regimen in Kenya (66).

Consistent with national projection for PrEP program, we anticipate that ~20% of women approached (~200 per clinic) will elect or qualify for PrEP and that a standardized initiation approach will be followed by algorithmic and simplified follow-up. PrEP medications will be provided by the Kenya national stock of antiretroviral as part of the national PrEP scale-up program. At each clinic, PrEP will be delivered by the usual trained clinic staff members (clinicians, nurses, etc.). We anticipate that women who choose to initiate PrEP will use it for up to 12 months, after which they will be re-evaluated.

***PrEP Discontinuation***

PrEP will be discontinued if (1) participant seroconverts (2) participants risk status changes (e.g. a partner who previously refused or was unable to take the self-test tests negative); (3) renal dysfunction with creatinine clearance <50ml/min; (4) client requests to stop; (5) sustained non-adherence; or (6) participant reports their HIV-positive partner has achieved sustained viral suppression. If a woman seroconverts during the study period, they will be linked with care at the nearest comprehensive care clinic to initiate ART.

***Support for PrEP adherence***

Adherence is a key driver of PrEP effectiveness. We will provide ongoing adherence counseling and support for women using PrEP. We think that women who choose to initiate PrEP as their preferred HIV prevention option will be more motivated to adhere to PrEP and that PrEP delivery models that systematically assess risk and optimize partner HIV status ascertainment will enhance adherence among women. Mobile phone-reminder systems are effective and recommended approach to support adherence to ART in HIV-infected persons (Chapter 5, 2016 Guidelines on Use of Antiretroviral Drugs for Treating and Preventing HIV infection in Kenya), and are routinely used by some clinics in Kenya to support routine care services including engagement with MCH and ART adherence in PMTCT. Extending experiences learned from the ART program, women who initiate PrEP at all facilities and feel comfortable about adherence support communication on their phone, will have an opportunity to receive brief mobile-phone communication to augment their adherence to PrEP.

***Visits***

For this program, where the goal is for real-world delivery of PrEP and seamless integration in routine service, formal visit schedule or retention efforts will reflect routine approaches used by MCH and FP clinics in Kenya. Women who initiate PrEP will be followed PrEP as per the Kenya national guidelines for PrEP (initiation, month 1, and then 3 monthly). We anticipate that women who choose to initiate PrEP will use it for up to 12 months, after which they will be re-evaluated.

***Seroconversion***

Persons identified as HIV infected in the program will be counseled and linked to HIV care immediately to start treatment.

**Safety**

Given the known high safety of antiretroviral medications for both treatment and prevention, the focus of the program on implementation is scale-up of PrEP delivery to at risk women rather than the medications themselves. Only data on serious adverse events (SAEs) that are unexpected and felt by treating clinicians to be related to PrEP will be collected. Importantly, data on kidney function, and infant growth data will be abstracted from program data tools.

***PrEP delivery operational tools***

Drawing on best practices identified through our team’s extensive work in PMTCT, male partner testing, and HIV program implementation in Kenya and extensively collaborative approaches, we will operationalize tools for PrEP delivery, partner self-testing, mhealth for PrEP adherence support, and other materials in MCH and FP settings as part of national PrEP scale-up program. These tools may include PrEP booklet, training modules and materials, posters, and assessment tools. We will work with clinics to harmonize data system MCH and FP and how to use clinic level data to improve services.

***Training and capacity building for PrEP implementation***

The program will work with NASCOP and Kisumu County authorities to support clinics’ readiness to deliver PrEP in a combination HIV prevention package in MCH and FP clinics. Health care workers in implementing MCH and FP clinics will be trained for competencies in the following domains:

- HIV risk assessment, counseling on PrEP initiation, discontinuation, adherence, interpretation of PrEP-related laboratory tests.
- Educational materials to sensitize women about PrEP in FP and MCH clinics.
- Standardized clinical tools for instructing women in FP and MCH clinics on delivering and performing male partner HIV saliva self-test.
- Integrating SMS systems to optimize adherence to PrEP.
- Interpretation and use of clinical-level data to monitor women on PrEP.

## LABORATORY METHODS

The program will be implemented through the usual normal routine MCH and FP clinic care system. Any laboratory monitoring will follow standard protocol implemented in MCH and FP clinics. For women who initiate PrEP, follow up will be conducted in accordance with the national guidelines for PrEP use (Chapter 11). Laboratory tests and schedule recommended by NASCOP for monitoring persons using PrEP include:

a) HIV testing: HIV testing is follow the national HIV testing services algorithm. HIV testing is required before PrEP initiation and then every three months. Initiation and monitoring of women who chose to initiate PrEP will follow the same protocol.

b) Creatinine testing: Creatinine testing is recommended at initiation and then annually. As part of the program goal to streamline clinic PrEP lab. systems for delivery of PrEP in MCH and FP clinics, point of care creatinine will be implemented to improve clinic flow efficiency. Women who have estimated creatinine clearance (CrCl) ≤50 ml/min will be discontinued from PrEP as soon as possible. In a subset of facilities with capacity for laboratory serum creatinine testing, we will conduct routine quality control and assurance procedures with the point of care machines per Kenyan National Quality Control Laboratory Standards.

c) Hepatitis B virus testing: Testing for Hepatitis B virus infection will follow the same procedures available in the implementing clinics as per NASCOP guidelines. At this time, not all clinics require the procedure or have the required materials to perform the procedure. Since this program aims to fit within the general clinic flow, Hepatitis B testing will vary clinic to clinic.

## RESEARCH COMPONENT

The impact of the national PrEP program on HIV infection among women will not only depend on PrEP uptake among at risk women but also on whether women who are prescribed PrEP use it appropriately. A research component will be nested in this program to evaluate adherence to PrEP at 4 facilities (4 MCH clinics and 4 FP clinics) using dried blood spots (DBS) for drug levels.

***Dried blood Drug Levels to Evaluate Adherence***

In all facilities, all women taking PrEP will have dried blood spot collected to test for tenofovir levels **up to a maximum of three visits** during the program. DBS are easy to implement, require less than 1 ml to process and it involves minimal risk no greater than risk encounter for figure prick for rapid HIV testing. Consistent with NASCOP projection for the first year of the national PrEP scale-up program, we anticipate that up 20% of women counsel (or approximately 200 at each clinic) will accept to use PrEP.

We will perform electronic-based consenting to the participants who are having DBS testing. After we have ensured that participants have read and understood the consent forms, we will ask them to append their signature on the tablet. Each participant will be offered a copy of the consent form to take home if they choose to. All the DBS samples will be stored in a freezer at -20^0^C. Some DBS samples for tenofovir levels will be shipped to Dr. Peter Anderson’s laboratory at the University of Colorado, Denver USA. This laboratory has extensive expertise in estimating PrEP adherence in clinical trials and programs. Ethical approval for shipment of samples will be obtained separately at the time of shipping. The shipped samples will be stored until the testing is done. By incorporating state-of-the-art adherence measurement with drug levels, we will have critical data to document adherence in real world settings and potentially identify PrEP delivery models that are better at attaining adherence. These data will help planning by NASCOP.

## DATA

The primary goal of this program is to integrate delivery of PrEP in standard clinical settings.

*Data collection*

All patient medical records will be captured through standard clinic data collection tools: FP and MCH registers; PrEP Card (a clinical tool developed by NASCOP to deliver PrEP in clinical settings), and tools developed for partner testing. We will use electronic (tablet)-based and paper based data forms to abstract some de-identified program data to assist in pogram quality improvement. Program de-identified data that may be abstracted may include records on: demographics; behavior-risk characteristics; partner self-testing; PrEP uptake; adherence to PrEP (from subset using PrEP); adverse events; and infant growth. All abstracted data will be uploaded onto REDCap® and maintained in a secure location. Internal quality control reports will be run on a monthly basis to monitor program progress, discussed with clinics, and NASCOP to improve implementation as the program comes to full scale.

*Outcomes*

Key outcomes will include: a) number of women at high risk of HIV initiating PrEP; b) adherence to PrEP; c) duration for which PrEP is used; d) women know partners HIV status; e) male partners tested for HIV; f) number of staff trained; g) adverse outcomes; h) new HIV infections averted, i) disability-adjusted life years saved, and j) incremental cost-effectiveness over standard MCH/FP care. We will characterize profiles of client in model of implementation that will further help in define women during seasons of elevated HIV risk.

*Analysis*

We will describe the performance of the two models program using relevant program data (e.g. PrEP uptake, adherence, and duration, partner HIV testing, and HIV infection rates as well as impact on clinic flow, time use, and costs). This will help define PrEP implementation model that is most efficiently reaching women who need PrEP during times of HIV risk.

Categorical variables will be detailed in tables as frequencies, and continuous measures will be summarized using means and standard deviations or medians and ranges, as appropriate. T-tests will be used to detect differences in mean levels of continuous variables and chi-square tests for dichotomous variables. Descriptive content will be inductively assembled to form explanatory accounts.

## HEALTH ECONOMICS

Data from the program will be used to conduct cost-effectiveness analyses to compare costs of offering and delivering PrEP in MCH and FP clinics. We will use micro-costing method to collect detailed information on resource use and costs focused on activities and inputs used to deliver services, including start-up activities (capacity strengthening, training and awareness raising), service delivery, lab monitoring, PrEP support, and PrEP) and HIV treatment costs averted. Mathematical models will be used to estimate the impact of the interventions on incident HIV infections and disability averted. Incremental cost effectiveness ratio of integrating the provision of PrEP through MCH and FP services will be compared across delivery approaches. Budget impact analysis will be conducted considering direct program costs to ensure that measurements of Kenyan MOH costs reflect the opportunity cost of resources used in delivering services. We will use guidelines to facilitate standardization of cost data collection and reporting to increase generalizability and transferability of results. Key deliverables are cost effectiveness of the different program delivery models, technical assistance and support, and scalable PrEP delivery strategies for at-risk young African women are developed.

## QUALITY ASSURANCE PROCEDURES

All program procedures will be implemented in accordance with the MoH protocols and guidelines. All data will be maintained in a secure location. Internal quality control reports will be run on a monthly basis to monitor program progress, discussed with clinics, and NASCOP to improve implementation as the program comes to full scale.

## DISSEMINATION

We will disseminate outcomes of M & E activities to national and international stakeholders and facilitate technical assistance for PrEP scale-up in other settings. Our finalized PrEP delivery model for women will be collated in stakeholder consultations and training programs coordinated by NASCOP. The delivery outcomes will include identification of efficient and cost-effective model of delivering PrEP in MCH and FP clinics, operationalization and adaptation of NASCOP PrEP delivery tools in MCH and FP clinics, counselling messages, flow charts/posters tailored for women in MCH and FP clinics and providers to work through prevention options, staffing requirements, and summaries of key scientific aspects learned during the implementation period. We will work with the national program in delivery of PrEP to optimize supply chain management of PrEP drugs, development and dissemination of provider guidelines and PrEP initiation/monitoring checklists, counselling guidelines, and clinician technical support. We will convene a national stakeholder meeting, including other parts of government and prevention providers, civil society, academia, and others to set the stage for next steps for full-scale implementation in MCH and FP clinics.

# HUMAN SUBJECTS CONSIDERATIONS

All program activities will be implemented through the standard routine MCH and FP clinic process. The protocol, implementation plan, data collection tools, and patient education materials will be reviewed and approved by the University of Washington Human Subjects Review Committee and the Kenyatta National Hospital-University of Nairobi Ethical Review Committee. Subsequent to initial review and approval, the responsible IRBs/ECs will review the program at least annually. We will provide oral consent for all program activities women screened with standard information describing programmatic rollout and procedures of the PrEP model implemented. For the research component, we will perform written consent for a subset of the women who elect to use PrEP for prevention to obtain dried blood spots for test drug levels for PrEP adherence.

## RISKS

For this roll-out program, where the goal is real-world delivery of PrEP and not medication themselves, the risks are expected to minimal and will be those expected in routine care. Participants may become embarrassed, worried, or anxious when talking about their personal history, their sexual history, ways to protect against infections passed during sex, and their test results. Partners may test positive, and knowing their status or the status of their partner may make them feel worried. If a participant or their partner finds out that they are positive, this may lead to disagreements or even physical or verbal abuse. It also may lead to economic risks such as loss of income. No safety or social harm concerns have been reported in studies of partner self-testing to date. Effort will be made to minimize the occurrence potential abuse by giving out HIV self-test kit to only women who feel comfortable delivering the kits to their male partner

## BENEFITS

Patients may benefit from ongoing access to enhanced HIV prevention services including PrEP to reduce the risk of HIV infection. All women will be provided with HIV prevention services, including HIV testing, PrEP, condoms, STI treatment, information on PMTCT, and ongoing support, including counselling and referral to other support services.

## TREATMENT FOR INJURY

Patients will be asked to inform the clinic staff if they feel they have been injured because of taking part in the program. Injuries may also be identified during laboratory testing, medical histories, and physical examinations. Treatment for adverse events will be provided through standard care services at the clinic. If treatment required is beyond the capacity of the clinic, the clinic staff will refer the patients to appropriate services or organizations that can provide care for the injury.

## INFORMED CONSENT

Informed consent will be conducted in a teired fashion: oral consenting for routine pogram activities that directly impact patient care, and written consent for the research procedures (i.e dried blood spots for drug levels) that will not directly be used for patient care but are important for overall program evaluation.

***Oral consenting for program activities***.

We will perform oral consenting for all women with standard consent information describing programmatic rollout and standard procedures for PrEP counseling and delivery (i.e. model of being implemented, PrEP prescription, partner self-testing, program data abstraction, and phone-based support for PrEP adherence). Staff conducting consenting will have the knowledge to cover any additional questions or concerns participants may have. All efforts will be made to ensure that women have a thorough understanding of the program and that their decision to be in the program is optional, and that not participating will not in any way affect their usual care. Women will be given a written copy of the consent information to take home with them if they choose to have it. Every effort will be made to protect patient privacy and confidentiality to the extent possible and only authorized person may have access to program records.

***Justification for oral consenting***

The goal of the program is to integrate PrEP delivery for HIV prevention as part of routine services offered to women receiving care in MCH and FP clinics in Kenya. Conducting oral consenting for non research procedures in this program will be consistent with standard procedures implemented in providing routine services in the busy MCH and FP clinics in Kenya including provision of PMTCT, HIV testing, STI screening, immunization, and cervical cancer screening. The proposed program procedures will be part of routine HIV prevention services offered to women attending MCH and FP clinics. The procedures represent no more than minimal risk and involves procedures for which consent would not normally be obtained outside the research context. The waiver of written consent will in no way affect the rights and welfare of the women receiving these services. We anticipate that utilizing oral consent will result in limited disruption of routine ANC/MCH services and will facilate seamless integration of PrEP counseling and delivery, which is a key intervention area for the Kenya MoH HIV Prevention Revolution Road Map⎯ to maximize efficiency in service delivery through integration (<http://hivhealthclearinghouse.unesco.org/sites/default/files/resources/kenya_hiv_prevention_revolution_road_map.pdf)>.

***Written consent for research activities***

In the research component, we will perform electronic consenting in a subgroup of women using PrEP to obtain dried blood spots for tenofovir levels to evaluate adherence to PrEP. After we have ensured that participants have read and understood the consent forms, women will be asked to append their signature on the tablet.

***Justification for non-assent for emancipated minors (15-17 year old women)***

The proposed program is integrating PrEP counseling and delivery as part of routine services to women of reproductive age attending MCH and FP clinics, including women aged 15 to 17 years who receive care from these clinics. It is expected that women 15-17 years who are already receiving routine services from antenatal and family clinics will be emancipated minors. All proposed program activities will be implemented according to national guidelines and current routine practices in MCH and FP clinics. The Kenyan National Guidelines for HIV Testing and Counselling references emancipated youth and adolescents and notes that “children may be tested with the consent of a parent or guardian, or may give their own if they are symptomatic, pregnant, married, a parent, or engaged in behavior that puts them at risk of contracting HIV,”. Similarly, the 2015 HIV Testaing Services (HST) Guidelines in Kenya state also reduced the age for HTS without the guardian/parent consent to 15 years including all emancipated minors who may be below 15 years of age and the guidelines define an emancipated minor as "A person who is not legally an adult but who, because he or she is married, a parent or otherwise no longer dependent on the parents." The National Reproductive Health Research Guidelines also details that “unless specific legal provisions exist, consent to participate in research should be given only by the adolescents”.

# PROGRAM RECORDS

## DATA OWNERSHIP

The proposed program is a collaborative effort between UW, KNH, NASCOP, and Kisumu County. The aforementioned institutions will jointly share ownership of the data. Authorship on publications, conference presentations, abstracts and other materials generated from this program will reflect contribution to design, execution and analysis of the program data.

## DATA RELEASE/SHARING POLICY

All data collected as part of this implementation program will be made available without cost with written request and agreement to the data sharing agreement after completion of primary analyses. The data sharing agreement will ensure commitments to:

- Using the data only for program evaluation purposes and without attempting to identify patients
- Securing the data using appropriate computer technology
- Destroying or returning the data after analyses are completed
- Restrictions on redistribution of the data to third parties
- Proper acknowledgement of the data resource.

## CONFIDENTIALITY

Every effort will be made to protect participant privacy and confidentiality to the extent possible. Personal identifying information will be retained at the local study site.

# LIMITATIONS

This is an implementation program. Quality improvement and program outcomes will be evaluated using abstracted program data which may be is prone to missingness. We will work with facilities to harmonize data management systems in MCH and FP clinics and we will regularly share clinic-level data that can be used to improve services in the clinics.

# TIMELINE

| Table 5: Program timeline | | | | | | | | | |
| --- | --- | --- | --- | --- | --- | --- | --- | --- | --- |
|  | 2016 | 2017 | 2017 | 2017 | 2017 | 2018 | 2018 | 2018 | 2018 |
|  | Sept. - Dec. | Jan. - March | April - June | July - Sept. | Oct. - Dec. | Jan. - March | April - June | July - Sept. | Oct. - Dec. |
| Protocol development |  | X |  |  |  |  |  |  |  |
| IRB/ERC applications |  | X |  |  |  |  |  |  |  |
| Staff hiring |  |  | X |  |  |  |  |  |  |
| Program implementation |  |  | X |  |  |  |  |  |  |
| Health Economics studies |  |  |  |  |  |  | X | X |  |
| Cost-effectiveness modeling |  |  | X | X | X | X | X | X |  |
| Manuscript writing |  |  |  |  |  |  |  |  | X |
| Dissemination |  |  |  |  |  |  |  |  | X |

# EXPECTED APPLICATION OF RESULTS

At the end of this program, we will have integrated PrEP at scale in routine public MCH and FP clinics in Kenya. Our findings of how effectively PrEP counseling and delivery is disseminated and implemented in MCH and FP clinics will inform national and county governments about to efficient target women who may benefit from PrEP. Cost-effectiveness data will guide policy makers’ decisions about allocation of resources towards anti-retroviral based HIV prevention interventions. Finally, we will operationalize national PrEP delivery tools including training modules, clinical delivery products and assessment tools that will support delivery of PrEP intervention at scale and will ensure that delivery continues at the national level.

# 14) REFERENCES

1. UNAIDS. *Global report: UNAIDS report on the global AIDS epidemic 2013.* Geneva2013.

2. Merson M, Padian N, Coates TJ, Aet al. Combination HIV prevention. *Lancet.* 2008;372(9652):1805-1806.

3. Kurth AE, Celum C, Baeten JM, Vermund SH, Wasserheit JN. Combination HIV prevention: significance, challenges, and opportunities. *Curr HIV/AIDS Rep.* 2011;8(1):62-72.

4. Kinuthia, J., Drake, A. L., Matemo, D., Richardson, B. A., Zeh, C., Osborn, L., … John-Stewart, G. (2015). HIV Acquisition During Pregnancy and Postpartum is Associated with Genital Infections and Partnership Characteristics: A Cohort Study. *AIDS (London, England)*, *29*(15), 2025–2033. <http://doi.org/10.1097/QAD.0000000000000793>

5. Drake, A. L., Wagner, A., Richardson, B., & John-Stewart, G. (2014). Incident HIV during Pregnancy and Postpartum and Risk of Mother-to-Child HIV Transmission: A Systematic Review and Meta-Analysis. *PLoS Medicine*, *11*(2), e1001608. <http://doi.org/10.1371/journal.pmed.1001608>

6. Lindegren ML, Kennedy CE, Bain-Brickley D, Azman H, Creanga AA, Butler LM, Spaulding AB, Horvath T, Kennedy GE (2012). Integration of HIV/AIDS services with maternal, neonatal and child health, nutrition, and family planning services. Cochrane Database of Systematic Reviews, Issue 9. Art. No.: CD010119. DOI: 10.1002/14651858.CD010119. available at: http://onlinelibrary.wiley.com/doi/10.1002/14651858. CD010119/abstract;jsessionid=E9C3C6212CF6DEC65E1D9BA76E52C6D4.f03t01

7. Thomson KA, Baeten JM, Mugo NR, Bekker L-G, Celum CL, Heffron R. Tenofovir-based oral preexposure prophylaxis prevents HIV infection among women. *Current Opinion in HIV and AIDS.* 2016;11(1):18-26.

8. Baeten JM, Donnell D, Mugo NR, et al. Single-agent tenofovir versus combination emtricitabine plus tenofovir for pre-exposure prophylaxis for HIV-1 acquisition: an update of data from a randomised, double-blind, phase 3 trial. *The Lancet Infectious Diseases.* 2014;14(11):1055-1064.

9. Baeten JM, Donnell D, Ndase P, et al. Antiretroviral Prophylaxis for HIV Prevention in Heterosexual Men and Women. *N Engl J Med.* 2012;367(5):399-410.

10. Murnane PM, Celum C, Mugo N, et al. Efficacy of preexposure prophylaxis for HIV-1 prevention among high-risk heterosexuals: subgroup analyses from a randomized trial. *AIDS.* 2013;27(13):2155-2160.

11. Choopanya K, Martin M, Suntharasamai P, et al. Antiretroviral prophylaxis for HIV infection in injecting drug users in Bangkok, Thailand (the Bangkok Tenofovir Study): a randomised, double-blind, placebo-controlled phase 3 trial. *The Lancet.* 2013;381(9883):2083-2090.

12. Thigpen MC, Kebaabetswe PM, Paxton LA, et al. Antiretroviral Preexposure Prophylaxis for Heterosexual HIV Transmission in Botswana. *N Engl J Med.* 2012;367(5):423-434.

13. Baeten J Heffron R, Kidoguchi L. et al. Near Elimination of HIV Transmission in a Demonstration Project of PrEP and ART. Conference on Retroviruses and Opportunistic Infections; Seattle, Washington; 2015.

14. McCormack S DDea. Pragmatic Open-Label Randomised Trial of Preexposure Prophylaxis: The PROUD Study. Conference on Retroviruses and Opportunistic Infections; 2015; Seattle, Washington.

15. Molina JM CC, Charreau I. et al. On Demand PrEP With Oral TDF-FTC in MSM: Results of the ANRS Ipergay Trial. Conference on Retroviruses and Opportunistic Infections; 2015; Seattle, Washington.

16. AVAC. *AVAC Report 2013: Research & Reality.* New York: AVAC;2013.

17. National AIDS and STI Control Programme (NASCOP). Guidelines on Use of Antiretroviral Drugs for Treating and Preventing HIV Infections in Kenya. 2016.

18. Karim SS, Karim QA. Antiretroviral prophylaxis: a defining moment in HIV control. *Lancet.* 2011;378(9809):e23-25.

19. Cohen J. AIDS research. Complexity surrounds HIV prevention advances. *Science.* 2011;333(6041):393.

20. Thirumurthy H, Masters SH, Mavedzenge SN, Maman S, Omanga E, Agot K. Promoting male partner HIV testing and safer sexual decision making through secondary distribution of self-tests by HIV-negative female sex workers and women receiving antenatal and post-partum care in Kenya: A cohort study. Lancet HIV [Internet]. Elsevier Ltd

21. AIDSinfo. Recommendations for Use of Antiretroviral Drugs in Pregnant HIV-1-Infected Women for Maternal Health and Interventions to Reduce Perinatal HIV Transmission in the United States. Bethesda, MD; 2015.

22. Benaboud S, Pruvost A, Coffie PA, Ekouévi DK, Urien S, Arrivé E, et al. Concentrations of tenofovir and emtricitabine in breast milk of HIV-1-infected women in Abidjan, Côte d’Ivoire, in the ANRS 12109 TEmAA Study, step 2. Antimicrob Agents Chemother. 2011;55(3):1315–7.

23. Mirochnick M, Best BM, Clarke DF. Antiretroviral pharmacology: Special issues regarding pregnant women and neonates. Clin Perinatol. 2010;37(4):907–27.

24. MURNANE, Pamela M. et al. “Pre-Exposure Prophylaxis for HIV-1 Prevention Does Not Diminish the Pregnancy Prevention Effectiveness of Hormonal Contraception.” *AIDS (London, England)* 28.12 (2014): 1825–1830. *PMC*. Web. 26 Feb. 2017.
