# Supplementary material for: Integrating preexposure prophylaxis delivery in routine family planning clinics: A feasibility programmatic evaluation in Kenya
Source: PLoS Med. 2019 Sep 3;16(9):e1002885. doi: 10.1371/journal.pmed.1002885 (PMC6719826; doi:10.1371/journal.pmed.1002885)
Supplement: S2 File — PrEP, preexposure prophylaxis. (DOCX) [file pmed.1002885.s003.docx]

**MINISTRY OF HEALTH
NATIONAL AIDS & STIs CONTROL PROGRAM**

## PrEP Rapid Assessment Screening Tool (RAST)

**Age:** _______________ **Sex:** _______________ **Date:** _________________

1. What is your HIV status*?* ***(if response is positive discontinue assessment else administer all questions)***

□Negative □Positive □Unknown □Unwilling to disclose

1. What is the HIV status of your sexual partner(s)?

□Negative □Positive □Unknown

**In the past 6 months**

1. Have you had sex without a condom with a partner(s) of unknown or positive HIV status?

□No □Yes

1. Have you engaged in sex in exchange of money or other favors?

□No □Yes

1. Have you been diagnosed with or treated for an STI?

□ No □ Yes

1. Have you shared needles while engaging in intravenous drug use?

□No □Yes

1. Have you been forced to have sex against your will or physically assaulted including assault by your sexual partner(s)?

□No □Yes

1. Have you used post exposure prophylaxis (PEP) two times or more?

□No □Yes

***Refer the client for further PrEP assessment at the health facility If:***

*HIV status of the sexual partner(s) is Positive or Unknown*

*Any* ***Yes*** to the screening questions

***Remarks*** *__________________________________________________________________________________________________________________________________________________________________________*

**Modified Rapid Assessment Screening Tool (RAST)**

Facility Name: Client ID: _ _ _ _ _- _ _- _ _ _ _ Date:

Affix RAST ID

Barcode here

Age(yrs): Sex: M F

Service Delivery Point: ANC PNC  FP OPD VCT TB Youth clinic

1. What is the HIV status of your sexual partner(s)

Negative  Unknown  Positive

**In the last 6 months, have you:**

**Yes No**

1. Had sex without a condom?
2. Engaged in sex in exchange of money or other favors?
3. Been diagnosed with or treated for an STI?
4. Been forced to have sex against your will or physically assaulted including assault by your sex partner?
5. Shared needles while engaging in intravenous drug use?
6. Used post exposure prophylaxis (PEP)more than twice?

8) Are you willing to consider PrEP?  Yes  No

1. If **NO**, what are some of the reasons you did not want to consider PrEP at this time? (tick all that apply)

Fear of side effects

Too many HIV tests

Taking drugs daily for a long time

Fear about effects on unborn baby

Partner known HIV Positive(Virally Suppressed)

Other (explain below)……………………………………...

Partner know HIV Negative

Fear that my partner/others will find out

I don’t think I’m at risk of acquiring HIV

Need to consult my partner

Fear of Intimate Partner Violence

| **CLINICAL RECORD VARIABLES** | | |  |  |  |  |  |  |
| --- | --- | --- | --- | --- | --- | --- | --- | --- |
| 10) | Marital status | Married | Widowed |  | Single | Divorced | Separated | Cohabiting |

**If entry point is a *FAMILY PLANNING* clinic, continue to question 11-12.**

| 11) | Currently using FP method | YES | | | No |  |
| --- | --- | --- | --- | --- | --- | --- |
| 12) | If Yes, Type Of FP | | | Injectable | Implant | OCP |
|  | |  | IUCD | | Condoms | Other…………………………….. |

**If entry point is an *MCH* clinic, continue to question 13.**

| 13) Currently pregnant (ANC) | Currently postnatal (PNC) |
| --- | --- |

**If *PREGNANT*, contine to question 14-16.**

1. Gestational age (weeks) …………………………………

| 15) | RPPR/VDRL | Reactive | Nonreactive | Not done/Unknown |  |  |
| --- | --- | --- | --- | --- | --- | --- |
| **If client is willing to consider PrEP, please counsel on PrEP.** | | | | | Counsellor Initials_________ | |
|  | | |  |  |  |  |
